# Evolutionary Analyses of GRAS Transcription Factors in Angiosperms

**DOI:** 10.3389/fpls.2017.00273

**Published:** 2017-03-02

**Authors:** Alberto Cenci, Mathieu Rouard

**Affiliations:** ^1^Bioversity InternationalMontpellier, France; ^2^CGIAR Research Programme on Roots, Tubers and BananasMontpellier, France

**Keywords:** GRAS gene family, transcription factors, plant evolution

## Abstract

GRAS transcription factors (TFs) play critical roles in plant growth and development such as gibberellin and mycorrhizal signaling. Proteins belonging to this gene family contain a typical GRAS domain in the C-terminal sequence, whereas the *N*-terminal region is highly variable. Although, GRAS genes have been characterized in a number of plant species, their classification is still not completely resolved. Based on a panel of eight representative species of angiosperms, we identified 29 orthologous groups or orthogroups (OGs) for the GRAS gene family, suggesting that at least 29 ancestor genes were present in the angiosperm lineage before the “Amborella” evolutionary split. Interestingly, some taxonomic groups were missing members of one or more OGs. The gene number expansion usually observed in transcription factors was not observed in GRAS while the genome triplication ancestral to the eudicots (γ hexaploidization event) was detectable in a limited number of GRAS orthogroups. We also found conserved OG-specific motifs in the variable *N*-terminal region. Finally, we could regroup OGs in 17 subfamilies for which names were homogenized based on a literature review and described 5 new subfamilies (DLT, RAD1, RAM1, SCLA, and SCLB). This study establishes a consistent framework for the classification of GRAS members in angiosperm species, and thereby a tool to correctly establish the orthologous relationships of GRAS genes in most of the food crops in order to facilitate any subsequent functional analyses in the GRAS gene family. The multi-fasta file containing all the sequences used in our study could be used as database to perform diagnostic BLASTp to classify GRAS genes from other non-model species.

## Introduction

Deep understanding of physiological mechanisms, biochemical reaction pathways or gene functions is required to improve plant performance, quality of production and environmental adaptability. However, most crops have features that constrain efficient experimentation (e.g., long generation time or large space needed for development). To circumvent these difficulties, physiological studies have been mostly performed on model plants chosen for their convenient experimental management. In particular, *Arabidopsis thaliana*, a small and short-lived plant belonging to the Brassicaceae family, is the model species most commonly used in plant research laboratories. Other plant species have been used as models to study more specific traits in taxonomic groups distantly related to *A. thaliana* or to study features not present in the model plant species. For instance, rice (*Oryza sativa*) has been extensively studied as a model for the Poales order (including other species having capital importance for human food security such as wheat, maize or sorghum) and *Medicago truncatula* as a model plant for symbiotic interactions between plant roots and nitrogen-fixing microorganisms.

As knowledge gained on model species can be transferred to crops of interest, it is fundamental to establish the orthology relationships between the genes of the model and the species of interest. In fact, even if the orthology is not a guarantee for function conservation, orthologous genes remain the best candidates for functional annotation transfer. Various automated approaches have been developed and proposed for identifying orthologous groups (OGs) that are particularly accurate for single-copy genes of small OGs (Trachana et al., 2011; Altenhoff et al., 2013). However, for genes belonging to multigene families, such as plant transcription factors, the definition of orthologous relationships is a more challenging task (Conte et al., 2008). The first requirement is the availability of the gene sequences for all the members of a given gene family in the studied species. The availability of complete quality genome sequences now makes it possible to perform unbiased and comprehensive comparisons.

In this study, we explored the evolution of the GRAS, a protein family that is involved in a wide range of different functions in plants. GRAS proteins appeared in land plants by lateral transfer from bacteria and underwent radiation in ancestors of bryophytes, lycophytes and higher plants (Zhang et al., 2012). Its name was derived from its first three identified members: GAI, RGA, and SCR (Pysh et al., 1999). It was suggested that GRAS proteins can act as transcription factors (Bolle, 2004; Hirsch and Oldroyd, 2009). The GRAS genes are almost all mono-exonic and encode for proteins with lengths between 360 and 850 amino acids (Supplementary Data 1). Several motifs have been recognized in their carboxyl (C-) termini: leucine heptad repeat I (LHR I), VHIID, leucine heptad repeat II (LHR II), PFYRE, and the SAW motifs. These five motifs constitute the GRAS domain. In contrast, the amino (N-) terminal part of GRAS proteins appears hypervariable (Pysh et al., 1999) and composed of intrinsically disordered domains that are involved in molecular recognition (Sun et al., 2011, 2012).

Several genome-wide analyses have been conducted on the GRAS family: in *A. thaliana* (Lee et al., 2008), in both *A. thaliana* and *O. sativa* (Tian et al., 2004), in *Brassica rapa* (Song et al., 2014), in *Pinus radiata* (Abarca et al., 2014), in *Prunus mume* (Lu et al., 2015), in *Populus trichocarpa* (Liu and Widmer, 2014), in *Solanum lycopersicum* (Huang et al., 2015), *Vitis vinifera* (Grimplet et al., 2016; Sun et al., 2016), in *Phyllostachys edulis* (Zhao et al., 2016), in *Ricinus communis* (Xu et al., 2016), in *Nelumbo nucifera* (Wang et al., 2016) and on a selected panel of plants, including *A. thaliana, O. sativa, Brachypodium distachyon, P. trichocarpa, Glycine max, Selaginella moellendorffii*, and *Physcomitrella patens* (Wu et al., 2014). The GRAS family has been usually divided into subfamilies such as DELLA, HAM, LS, LISCL, NSP2, PAT1, SCR, SCL3, and SHR. However, all these studies reported a different number of subfamilies (8–13) defined on their respective phylogenetic results.

In order to establish a consistent framework for the classification of GRAS members in angiosperm species, we performed a genome-wide study of the GRAS genes by reconstructing their evolutionary history and defining orthologous groups (Kuzniar et al., 2008) in the monocots' and dicots' lineages. An orthologous group includes all the gene family members derived from a common ancestor gene existing before the radiation of the considered species. In this study we considered the split between monocots and dicots as the reference to define the frameshift of orthology relationships. We selected eight species that we considered representative of the angiosperms because of their plant model status, the absence of lineage-specific whole genome duplications and/or their critical position in angiosperms, balanced between monocots and dicots. For monocots: *Musa acuminata* (Zingiberales), *Phoenix dactylifera* (Arecales) and *O. sativa* (model species and Poales); for dicots: *A*. *thaliana* (model species), *V. vinifera, Theobroma cacao* (rosids) and *Coffea canephora* (asterid); and *Amborella trichopoda* as the basal angiosperm and outgroup for monocots and dicots phylogenies. Here we identify and define orthogroups that are then individually discussed.

## Materials and methods

### Data sets

An initial cluster set of 413 protein sequences annotated in genomes of *Arabidopsis thaliana* (Kaul et al., 2000), *Vitis vinifera* (Jaillon et al., 2007), *Theobroma cacao* (Argout et al., 2011), *Coffea canephora* (Denoeud et al., 2014), *Phoenix dactylifera* (Al-Mssallem et al., 2013), *Oryza sativa* (Sequencing Project International Rice Genome, 2005), *Musa acuminata* (D'Hont et al., 2012), and *Amborella trichopoda* (Albert et al., 2013) were retrieved from GreenPhylDB (http://www.greenphyl.org/) (Rouard et al., 2011). All sequences were revised, compared to GenBank protein databases and, when needed, corrected and integrated with previously published studies to produce a subset of 397 validated sequences (Supplementary Data 1 and Supplementary Table 1).

### Detection of orthologous groups (OGs) and their organization in subfamilies

The manually curated inference of OGs was performed on the basis of reciprocal BLASTp analyses, as described in Cenci et al. (2014). Briefly, a BLASTp analysis was performed for each GRAS sequence on protein databases of the studied species; for each species, proteins with best scores were grouped and those belonging to the OG confirmed by reciprocal BLASTp.

In the case of species missing representatives in one or more OG, BLASTp analyses were performed on “nr” protein database (All non-redundant GenBank CDS translations + PDB + SwissProt + PIR + PRF excluding environmental samples from WGS projects) to look for the presence of OG members in wider taxonomic groups. In order to be consistent with GRAS classification in previous studies and to have a more detailed picture of the GRAS family in angiosperms, subfamilies were established. A subfamily is usually defined as a group of sequence displaying a significant degree a sequence similarity, regardless of phylogenetic events. Here, a subfamily is considered with regards to the orthogroups it contains. Consequently, as higher classification rank of OG, a subfamily can include one to several OGs. The subfamily names were defined based on the name assigned to the first described gene. The names of OGs included in a same subfamily were differentiated by a hyphen and a number (Table 1). Hyphens and numbers were not added in non-assembled OGs (in these cases a subfamily included only one OG).

**Table 1:** **Numbers of GRAS genes of the studied species assigned to each OG**.

**Orthologous group**	**Amb**	**Ma**	**Pd**	**Os**	**Vv**	**Tc**	**At**	**Cc**	**Tot**
OG-SCR-1	1	3	2	2	1	1	1	1	12
OG-SCR-2	2	1	2	1	1	1	1	1^*^	10
OG-SCR-3	1	−^a^	1	1^*^	1	1	−^b^	1	6
OG-SHR-1	1	3	2	2	1	1	1	1	12
OG-SHR-2	1	3	2	−^c^	1	1	−^b^	1	9
OG-SCL32-1	1	3	2	1	1	1	1^*^	1	11
OG-SCL32-2	1	1	1	1	1	1	−^b^	2	8
OG-NSP1	1	1	2	1^*^	1	1	1	1	9
OG-LS	1^*^	4	4	2^*^	1	1	1	1	15
OG-SCL4/7	1	3	2	1	1	1	2	1	12
OG-NSP2-1	1	1	2	1	1	1	1	1	9
OG-NSP2-2	1	−^a^	1	2	1	−^d^	−^b^	1^*^	6
OG-NSP2-3	1	−^e^	−^e^	−^e^	3	2	−^b^	2	8
NSP2-Amb	2	−	−	−	−	−	−	−	2
OG-HAM-II	1	7	4	5	2	2	3	2	26
OG-HAM-I	1	3	2	−^c^	1	1	1	1	10
OG-DELLA-1	1^*^	4^*^	2	1	2	2^*^	5	2^*^	19
OG-DELLA-2	1	1	2	2	1	1	−^b^	1	9
OG-PAT-1	1	3	2	3	3	3	4	2	21
OG-PAT-2	1	4	2	1	1^*^	1	−^f^	1	11
OG-PAT-3	1	3	1	1	2	1	1	1	11
OG-PAT-4	−	7^*^	2	2^*^	1	1	1	1	15
OG-SCL3	1	3	3	9^*^	3^*^	3	1	2	25
OG-RAD1-1	1	1	1	1^*^	2	2	−^b^	2	10
OG-RAD1-2	1	−^a^	1	−^c^	1	1	−^b^	−^g^	4
OG-RAM1	1^*^	1	1	1	1	1	−^b^	1	7
OG-DLT	1	3^*^	2	1^*^	1	1	1	1	11
OG-SCLA	1	2	2	1	1	1	−^b^	2	10
OG-SCLB	4^*^	−^a^	5	−^c^	1^*^	3	−^b^	5^*^	18
OG-LISCL	1	7	4	13^*^	11^*^	7	7	11^*^	61
Total	34	72	59	56	49	44	33	50	397

* *Indicates presence of remnant(s) in the genome*.

a *Missing in M. acuminata*.

b *Missing in Brassicales*.

c *Missing in Poales*.

d *Missing in Malvales*.

e *Missing in monocots*.

f *Missing in Brassicaceae but present in other Brassicales*.

g *Missing in C. canephora*.

*Os, Oryza sativa; At, Arabidopsis thaliana; Amb, Amborella trichopoda; Ma, Musa acuminata; Pd, Phoenix dactylifera; Vv, Vitis vinifera; Tc, Theobroma cacao; Cc, Coffea canephora*.

### Phylogenetic analyses

A phylogenetic reconstruction was performed for each OG, using all the GRAS sequences identified for each OG in the eight studied species. The phylogenetic analysis of the complete GRAS family was performed using all the member sequences of eight studied species and also with *A. trichopoda, V. vinifera*, and *P. dactylifera*. For both, protein sequences were aligned with MAFFT program (Katoh and Standley, 2013) via the EMBL-EBI bioinformatics interface (Li et al., 2015) using default parameters. Conserved blocks were extracted from the alignments with Gblocks (http://molevol.cmima.csic.es/castresana/Gblocks_server.html) (Castresana, 2000). The analysis was performed by allowing: (i) smaller final blocks, (ii) gap positions within the final blocks, and (iii) less strict flanking positions. Phylogenetic trees were built with PhyML (Guindon et al., 2009) available at http://phylogeny.lirmm.fr/ (Dereeper et al., 2008) using an LG substitution model and an Approximate Likelihood-Ratio Test (aLRT) as statistical tests for branch support. Phylogenetic trees were visualized with MEGA6 (Tamura et al., 2013) and iTOL (http://itol.embl.de/) (Letunic and Bork, 2016).

### Conserved domain analysis

All OGs were scanned separately with MEME search using MEME v4.10 up to 10 domains (Bailey et al., 2015). One representative corresponding to the sequence with the smallest genetic distance in the gene tree for each OG was selected and a new MEME search was applied with domain limit up to 30.

### Syntenic analyses

The location of the genes in syntenic blocks was retrieved using SynMap program (http://genomevolution.org/CoGe/SynMap.pl) obtained using a default parameter (Lyons et al., 2008) except for *C. canephora* which was studied with the Coffee Genome Hub (http://coffee-genome.org/syntenic_dotplot) (Dereeper et al., 2015).

## Results

**Figure 1:**
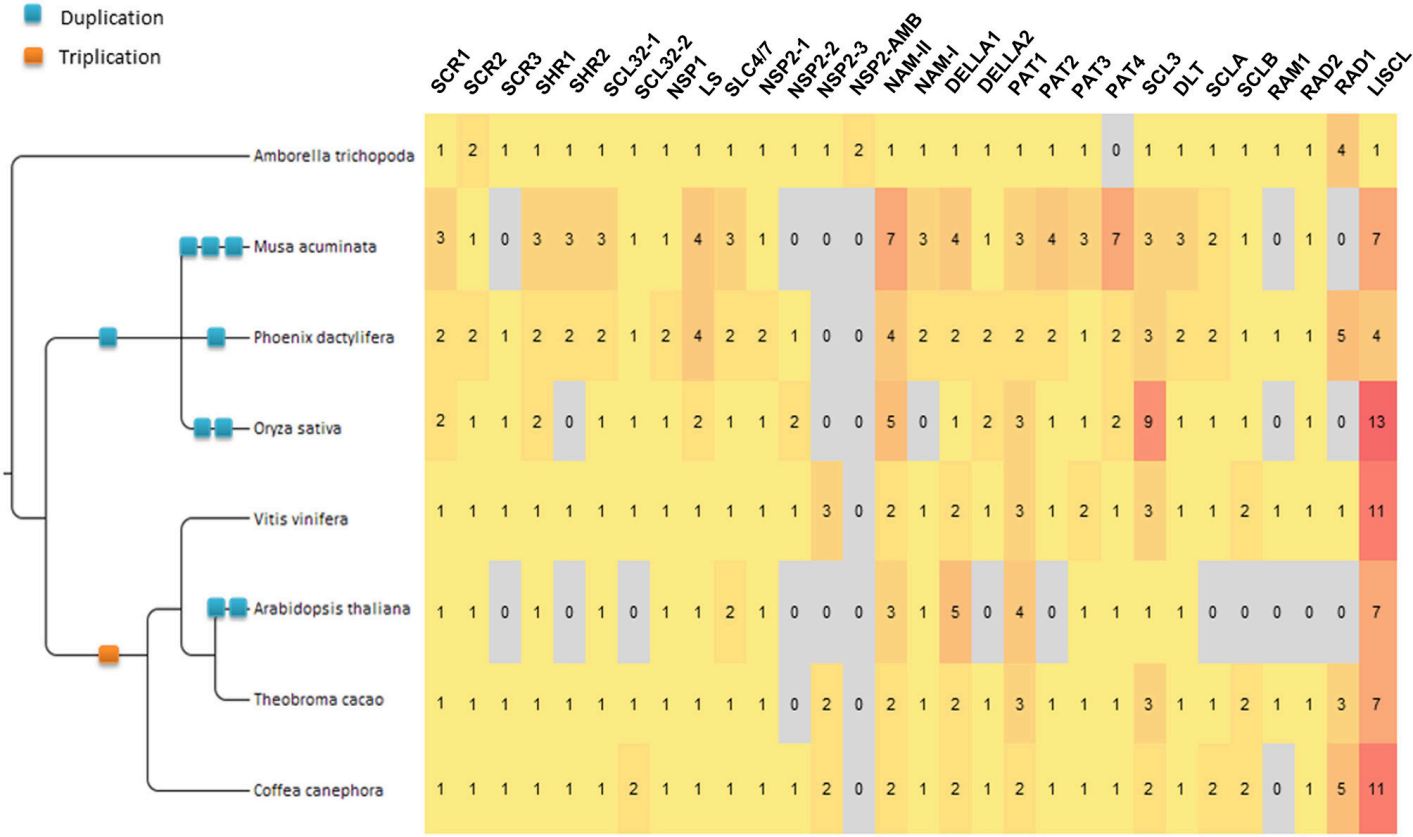
**GRAS Gene distribution by orthogroup in 8 plant species**. Numbers in the matrix represent the number of genes by OG specified in the header (with the exception of NSP2-AMB). The gradient color from yellow to red illustrates the abundance of genes. The WGD involving the ancestor of all the studied dicot species (γ event, hexaploidization) is indicated by an orange square.

All protein sequences annotated as GRAS in eight angiosperm species were manually curated and, where necessary, gene structure corrections of the sequences were performed. A total of 397 sequences were retained for this study (Supplementary Data 1 and Supplementary Table 1). Twenty-nine orthologous groups (OGs) were defined for the GRAS gene family in our panel of species (Table 1; Figure 1; Supplementary Table 1). The GRAS sequences used in this study were stored in GreenPhylDB (http://www.greenphyl.org/cgi-bin/custom_family.cgi?p=id&custom_family_id=158789) (Rouard et al., 2011) and organized in OGs and in subfamilies. Specific gene losses were observed for some OGs, especially involving *A. thaliana* for which genes were absent in 12 OGs.

**Figure 2:**
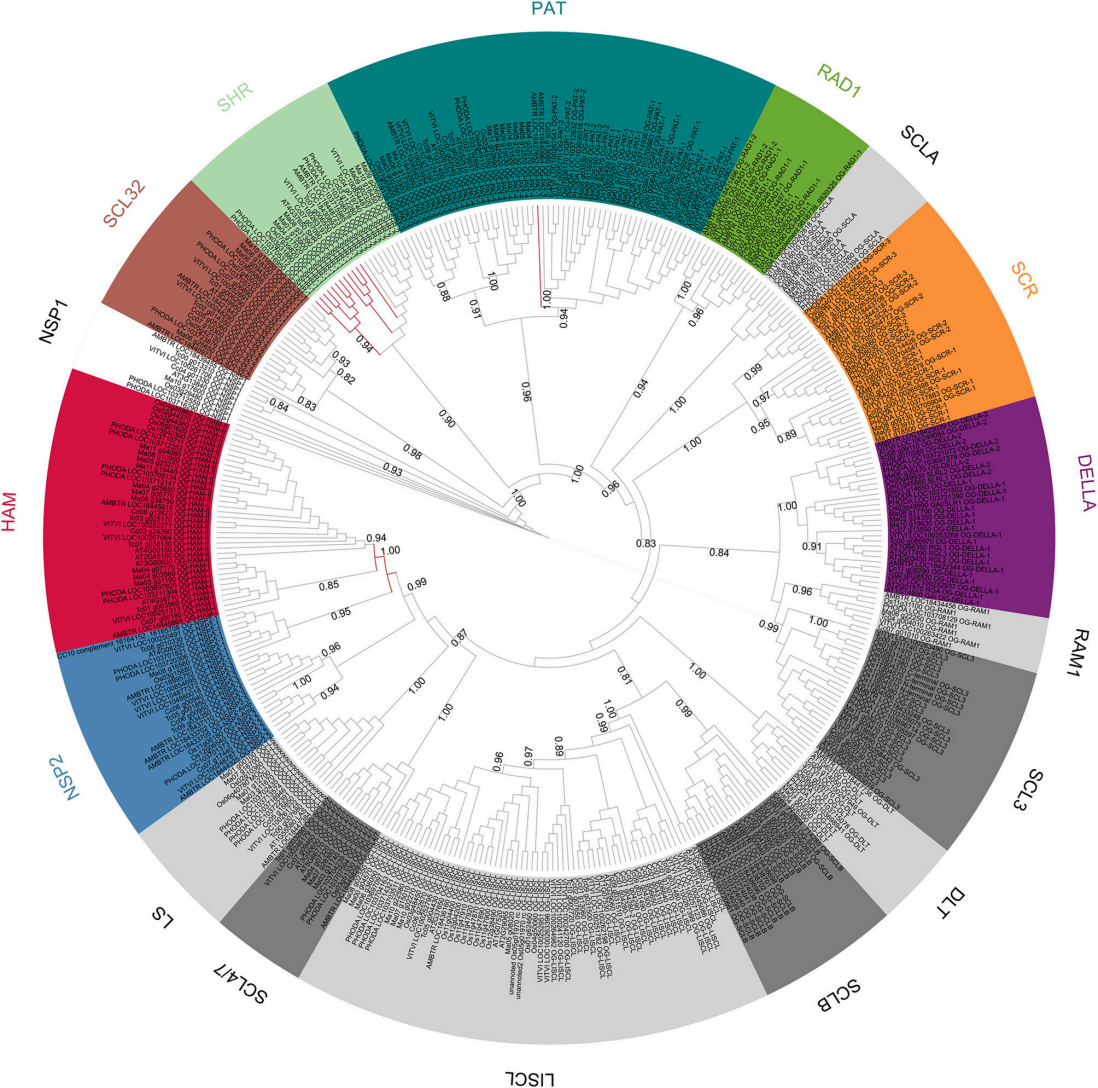
**Unrooted ML phylogenetic tree based on GRAS sequences from *Amborella trichopoda*, *Phoenix dactylifera* and *Vitis vinifera*, *Musa acuminata*, *Oryza sativa*, *Arabidopsis thaliana Theobroma cacao* and *Coffea canephora***. Branch support is based on aLRT score. The background colors differentiate subfamilies including more than one OG. OGs not grouped in subfamilies are differentiated by gray tones. Branches that were inconsistent with defined orthogroups were colored in red.

A phylogenetic tree was built with the 397 GRAS protein sequences, belonging to the eight species studied (Figure 2). The phylogenetic tree was computed with 151 aligned positions, all belonging to the GRAS domain. Overall, the results were consistent with the OG definition but few exceptions were observed. HAM and NSP2 Subfamilies were not resolved. The HAM subfamily (OG-HAM-I and OG-HAM-II clusters) were included in a cluster with OG-NSP2-1 (aLRT support only 0.5) whereas the OG-NSP2-2 and OG-NSP2-3 formed another cluster with two Amborella sequences unassigned to any OG. Concerning the subfamily SHR, OG-SHR-2 sequences formed a cluster whereas OG-SHR-1 was not resolved as the sequences split in one monocot and one dicot cluster with an isolated Amborella sequence. The branch support of the most of the clusters is low (aLRT spanning between 0.74 and 0.94). Finally, the Amborella sequence assigned to OG-PAT-1 was not included in its respective cluster.

**Figure 3:**
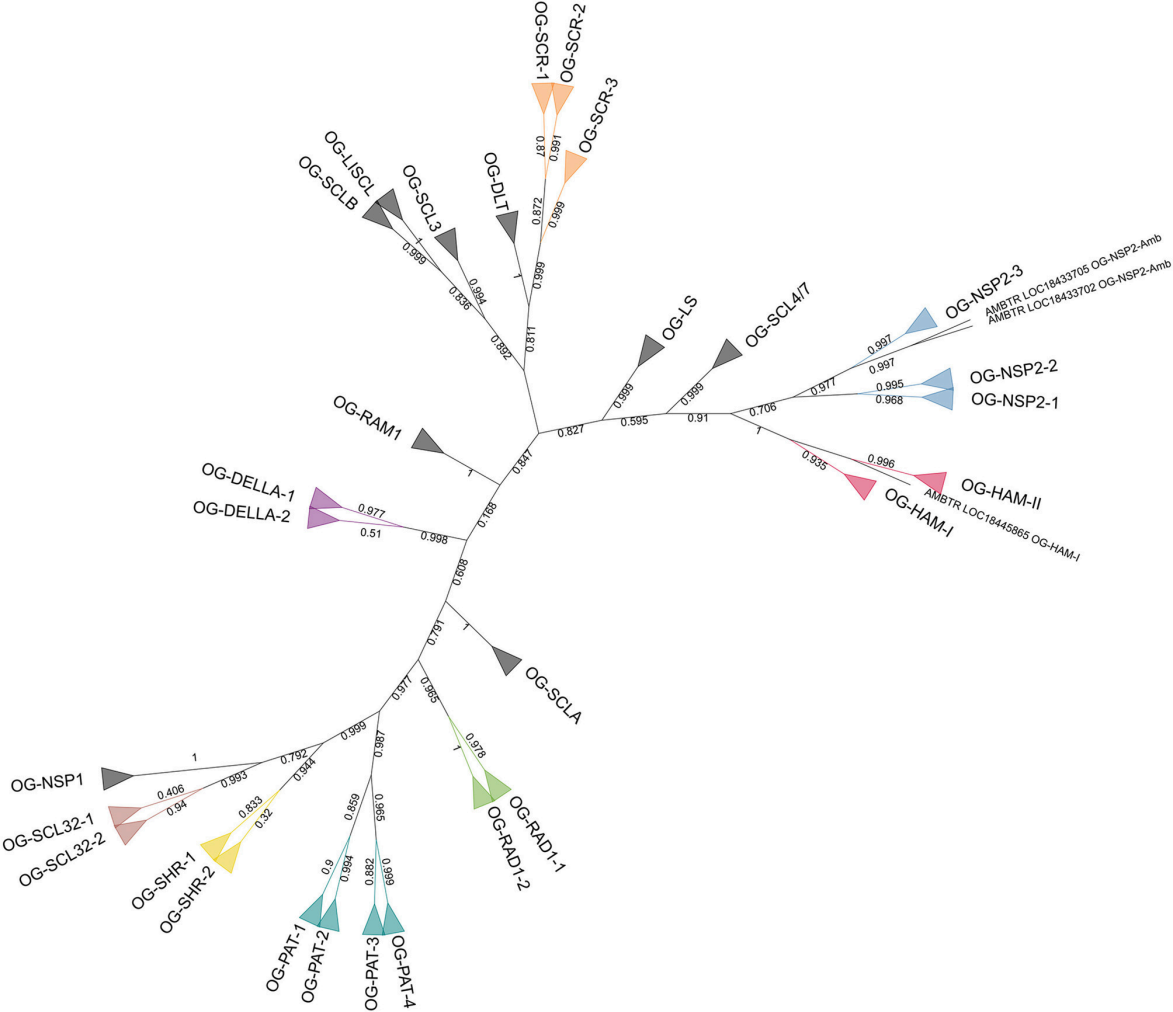
**Unrooted ML phylogenetic tree based on GRAS sequences from *Amborella trichopoda*, *Phoenix dactylifera*, and *Vitis vinifera***. Branch support is based on aLRT score. Clades containing genes assigned to a same OG are collapsed. The colors differentiate subfamilies including more than one OG. OGs not grouped in subfamilies are in black. Individual sequences not associated to any OG are not collapsed.

Another phylogenetic tree was built with the 142 GRAS protein sequences of *A. trichopoda, V. vinifera*, and *P. dactylifera* (Figure 3). The phylogenetic tree was computed based on 180 aligned positions, all belonging to the conserved GRAS domain. The results of the analysis were highly consistent with the initial OG definition (with the only exception of the *A. trichopoda* sequence included in the OG-HAM-I that was not resolved in the correct cluster), showing 29 clusters including the respective sequences of 29 OGs and clustering all the OG included in subfamilies (Figure 2).

For increased resolution, unrooted phylogenetic trees including all eight of the species were built for each OG and for each subfamilies (Supplementary Data 2; Supplementary Figures 1–38). The phylogenetic analyses were performed with a number of positions (belonging to both the GRAS domain and to the *N*-terminal region) spanning between 255 and 520 for the OGs and between 170 and 368 for the subfamilies.

We also analyzed OG sequences by MEME search and almost all the conserved motifs were located in the GRAS domain. The analysis performed with a representative of all OGs did not detect any conserved regions outside of the GRAS domain. However, by investigating OGs individually, 19 OGs displayed one or two conserved motifs in the *N*-terminal region (Table 2).

**Table 2:** **Conserved motifs identified by MEME software among defined gene clusters**.

**OG**	***p*-value**	**Sequence**	**Exceptions/missing**
OG-SCR-1	1.4e-093	MDDTAATAWIDGIIRDIIHSS	None
	4.2e-217	VSIPQLIHNVREIIHPCNPNLAAILEYRLRSLM	None
OG-SHR-1	1.1e-097	CHHFYMDEDFFSSSSSKHYHP	None
OG-SCR32-1	8.0e-053	HQIGPCLDLTMNKNQIHRTRPWPGFPTSK	*A. thaliana, O. sativa, M. acuminata* (1) and *A. trichopoda*
OG-SCL4/7	2.6e-311	MAYMCTDSGNLMAIAQQVIKQKQQQQQQQQQQQQQQQ	None
	3.8e-103	EFDSDEWMESLMGGGDAEESDNM	None
OG-NSP2-1	1.8e-031	DDFHDLIESMMCD	None
OG-HAM-I	6.3e-049	NNNNHFNCNLCYEPTSVLDPHLSPSPVTE	*A. thaliana*
OG-DELLA-1	1.2e-429	DCGM**D**E**LLA**VL**GYKVRSSD**MAD**VA**QK**LE**QLE**MV**MGNSQEDN	None
	5.3e-299	SHL**AS**DT**VHYNPSDLSTWVDSMLSELNP**PPPPIPPPPPIPP	*A. thaliana* (2)
OG-DELLA-2	1.0e-079	EI**D**G**LLA**GA**GYKVRSSD**LHH**VA**HRLER**LE**TA**MV**NA	*O. sativa* (2)
	6.0e-057	**AS**EA**VHYNPSDLTSWVDSMLSELNP**	*O. sativa* (2)
OG-DELLA-1,2	6.8e-531	QDCGM**D**E**LLA**VL**GYKVRSSD**MAD**VA**QK**LE**QLE**MV**MGNAQED	*O. sativa* (2)
	1.6e-405	L**AS**DT**VHYNPSDLSTWVDSMLSELNP**PPP	*O. sativa* (2)
OG-PAT-1	3.6e-159	FQQNSQSYPSDQHHSPDNTYGSPISGSC	*M. acuminata* (1) and *A. thaliana* (1)
	3.7e-170	TDDPNDLKHKLR**E**L**E**TAMLGP	None
OG-PAT-2	1.1e-108	Q**E**I**E**TVLMAPDTDEPTTSTNVECDENKQPQLTKQRSRTWTH	None
OG-PAT-3	1.5e-101	IDYDEDEMRLKLQ**E**L**E**QALLNDNDEDLYD	None
OG-PAT-4	9.1e-105	GGILKRSLTEMERQQQQQQQQ	None
	7.9e-094	LQ**E**L**E**KQLLDDDDEE	None
OG-PAT-1234	1.4e-421	ITYDENDMKHKLQ**E**L**E**TAMMGDDDDDE	*M. acuminata* (1) and *A. thaliana* (1)
OG-SCL3	4.4e-127	QQDDGSSSVTSSPLQFFSLMSLSPGTGSP	*O. sativa* (8), *P. dactylifera* (1), *V. vinifera* (1) and *T. cacao* (1)
OG-DLT	4.9e-074	MGTQRLDLPCSFSRK	None
OG-RAM1	4.3e-033	KGKGQSPLHKVFNSPNNQYMQ	*A. trichopoda*
OG-RAD1-1	4.6e-079	NRNGSTNSTNSLPRLHFRDHIWTYKQRYLAAEAMEEAAAAM	None
OG-RAD1-2	1.5e-011	SFNHDTAIRRFCPARIEQEQ	None
	4.7e-008	PPSLAASEEDEFVDSFINMDWCDDYDND	None
OG-SCLA	2.1e-035	DVCEGKFFGLLQARERMLKVDPKRKGMED	*C. canephora* (2)
OG-SCLB	5.4e-171	NYRSSHGRLCGEKENEPTDGVTYPTGGGDELSTEEVIRIAGAHYVYMGTH	*C. canephora* (1) and *T. cacao* (1)

*Bold characters are used to highlight conserved amino acids in motifs identified in OGs classified in the same subfamily. In subfamily DELLA, where two conserved motifs were detected, second motif amino acids are underlined. In the last column are reported the species (and the number of relative sequences) where the motif was not detected*.

Hereafter, we described all subfamilies and orthogroups including reference and comparison to previously published results.

### Scarecrow (SCR) subfamily

The SCR genes are involved in the *A. thaliana* bundle sheath and mesophyll cell fate (Cui et al., 2014) and contributing to ground tissue radial patterning in both embryonic root and shoot (Di Laurenzio et al., 1996; Wysocka-Diller et al., 2000; Heidstra et al., 2004).

The scarecrow subfamily is composed of three orthologous groups (OG-SCR-1, -2, and -3; Table 1; Figure 2). Phylogenetic analyses performed with all sequences of these three OGs distinguished three clusters, perfectly consistent with the OG composition (Supplementary Figure 4). SCR (AT3G54220) and SCL23 (AT5G41920) genes from *Arabidopsis* are included in these OGs. All OG-SCR-1 sequences are characterized by an intron in a conserved position inside the GRAS domain, whereas the members of the two remaining groups have the typical mono-exonic structure. Two adjacent conserved motifs (21aa and 33aa, respectively) were found in the *N*-terminal region of OG-SCR-1 only (Table 2). In our global phylogenetic analyses, the SCR subfamily does not appear to have any close relationships with any other GRAS OG (Figures 2, 3).

### Short root (SHR) subfamily

In *A. thaliana*, the SHR gene (AT4G37650) is known to be involved in the bundle sheath and mesophyll cell fate (Cui et al., 2014) by regulating the expression of SCR and SCL23. The SHR subfamily is composed of two OGs. Members of OG-SHR-2 were absent in *O. sativa* and *A. thaliana* and were also missing in the Poales and Brassicales orders (Table 1). Phylogenetic analysis clearly separates the members of the two OGs (Supplementary Figure 7). All genes are mono-exonic but the *V. vinifera* member of OG-SHR-1 (VITVI_LOC100263197) contains an intron. A 21aa-conserved motif was found in OG-SHR-1 proteins only (Table 2).

### Scarecrow-like 32 (SCL32) subfamily

Since no function was assigned to the member of this subfamily, the name Scarecrow-like 32 (SCL32), derived from the *A. thaliana* member (AT3G49950), was chosen as OG identifier. In the phylogenetic analysis performed with all the members of the subfamily, two major clusters consistent with the defined OGs were identified in the phylogenetic analysis (Supplementary Figure 10). A 29aa-conserved motif was identified in almost all members of OG-SCR32-1 (Table 2), with the exception for *A. thaliana, O. sativa*, and *A. trichopoda*.

### Nodulation signaling pathway 1 (OG-NSP1)

The function of a member of this OG was first characterized in *Medicago truncatula* (Supplementary Figure 35) where it regulates the expression of nodulation factors (Smit et al., 2005). However, the members of this group play a role also in non-legume species. Liu et al. (2011) showed the role of NSP1 (and NSP2) in the biosynthesis of strigolactone in *M. truncatula* and *O. sativa*. The OG-NSP1 includes the *A. thaliana* member AT3G13840 (SCL29) and at least a member for each analyzed species (Table 1; Supplementary Figure 11). Phylogenetic relationships were shown among OG-NSP1, SHR and SCL32 OGs based on *A. thaliana* and *O. sativa* member sequences (Tian et al., 2004; Lee et al., 2008; Wu et al., 2014). These relations were confirmed in Figure 2. A 14aa-motif was found conserved in all species except for *A. trichopoda* and *P. dactylifera* sequences (Table 2).

### Lateral suppressor (OG-LS)

The first gene (Ls) of this OG was isolated in *S. lycopersicum* (Schumacher et al., 1999; Supplementary Data 3). The OG-LS includes two genes for which the function was characterized in the studied species: Lateral suppressor (LAS) in *A. thaliana* (AT1G55580) (Greb et al., 2003) and Monoculum1 in *O. sativa* (Li et al., 2003). In both plants, the orthologs control the formation of lateral shoots during vegetative development. In *O. sativa* a paralog is also present whereas in *M. acuminata* and *P. dactylifera* the ancestral gene was amplified with four members found in both genomes (Table 1). The phylogenetic analysis placed the *A. trichopoda* gene inside the dicot cluster, close to the *A. thaliana* gene (Supplementary Figure 12). Since both the *A. trichopoda* and *A. thaliana* genes have long branches, this unexpected position was suspected as a long-branch attraction (LBA) artifact. To test this hypothesis, the analysis was repeated removing the *A. thaliana* sequence. The resulting phylogenetic tree shows *A. trichopoda* to be no longer included in the dicot cluster (Supplementary Figure 13) suggesting the LBA as likely explanation of the anomalous position of the *A. trichopoda* gene.

### Scarecrow-like 4 and 7 (OG-SCL4/7)

Since no function was assigned to the member of this OG, its name (OG-SCL4/7) name was derived by the two *A. thaliana* paralogs Scarecrow-like 4 (AT5G66770) and Scarecrow-like 7 (AT3G50650) included in this OG. A *Populus euphratica* ortholog (PeSCL7) was found overexpressed during the early stage of an induced severe salt-stress. Transgenic *A. thaliana* plants overexpressing this GRAS gene showed increased tolerance to salt and drought stresses (Ma et al., 2010). It is worth noting that, in contrast to other OGs, the first 19 amino acids of the OG-SCL4/7 members are highly conserved among all the species (MAYMC[A/T]DSGNLMAIAQQ[V/L]I) which corresponds to a coiled coils structures (http://embnet.vital-it.ch/software/COILS_form.html). This region was part of a 37aa-motif detected by MEME analysis along with another motif 23aa long (Table 2).

### Nodulation signaling pathway 2 (NSP2) subfamily

The function of a member of this OG was first characterized in *Medicago truncatula* where it regulates the expression of Nodulation factors (Smit et al., 2005; Supplementary Data 3). The OG-NSP2-1 includes the *A. thaliana* member AT4G08250 (SCL26) and at least one member for each analyzed species (Table 1). The OG-NSP2-1 also contains the *Nicotiana tabacum* gene NtGRAS-R1, a topping responsive gene (Xu F. et al., 2015).

The OG-NSP2-2 includes genes from *O. sativa, P. dactylifera, V. vinifera* and *C. canephora*, whereas members are missing in Brassicales and Malvales, as well as in *Musa*. The *O. sativa* gene DIP1 (DELLA Interacting Protein 1, Os12g06540) that interacts with SLR1 gene in rice and is involved in the arbuscular mycorrhizal symbiosis (Yu et al., 2014) is included in this OG.

OG-NSP2-3 includes members from most of the analyzed dicots (*V. vinifera, T. cacao, C. canephora*) and *A. trichopoda* but no members were found in monocots (Table 1). Finally, two additional close *A. trichopoda* sequences do not have any close sequences in monocots or dicots. Even if the *A. trichopoda* specific sequences are located in tandem, identity and similarity between them is reduced (51 and 66%, respectively), suggesting they originated from an ancient tandem duplication event. A short conserved motif (13aa) was detected in OG-NSP2-1 sequences (Table 2).

### Hairy meristem (HAM) subfamily

Hairy meristem mutation was described in *Petunia hybrida* (Stuurman et al., 2002; Supplementary Data 3), where it is essential and specific for maintaining the shoot apical meristem. In *A. thaliana*, three close and redundant genes (Atham1, 2, and 3; AT2G45160, AT3G60630 and AT4G00150, respectively) were described along with a fourth, more distantly related homolog (Atham4, AT4G36710) (Engstrom, 2011). Two OGs were recognized, including these genes, and are named based on classification in Engstrom et al. (2011): OG-Ham-II which contains the most of the Ham genes (included the *P. hybrida* one) and OG-Ham-I. One *A. trichopoda* sequence is included in OG-Ham-II (LOC18445615), whereas a second Ham gene in Amborella (LOC18445865) appears to belong to the OG-Ham-I (Table 1). A 29aa-long conserved motif was detected in OG-HAM-I sequences (Table 2).

### DELLA subfamily

DELLA proteins are repressors of the Gibberellin responses and take their name from the presence of the conserved DELLA domain in the sequence of the *N*-terminal region. In *A. thaliana*, five members of this family were recognized: RGL1, 2, 3, RGA1, and GAI (AT1G66350, AT3G03450, AT5G17490, AT2G01570, and AT1G14920, respectively) (Silverstone et al., 1998; Park et al., 2013). All five genes are part of the OG-DELLA-1 as well as the *O. sativa* Slender rice 1 gene (SLR1, Ikeda et al., 2001). In addition to the above cited genes, other DELLA-like genes are part of the OG-DELLA-2. It is worth noting that both *O. sativa* genes on OG-DELLA-2, described as Slender like SLRL1 and SLRL2 (Itoh et al., 2005), corresponding to Os01g45860 and Os05g49930 respectively, lost their functional DELLA protein domain even though their inclusion in this OG is very well supported. A conserved motif corresponding to the DELLA domain was found in almost all sequences of both OGs and it was detected again when the sequences of both OGs were analyzed together (Table 2).

### Phytochrome a signal transduction (PAT) subfamily

PAT1 gene was shown to be a positive regulator in transduction of Phytochrome A signal in *A. thaliana* (Bolle, 2004). Four OGs are included in the PAT subfamily. OG-PAT-1 includes the *O. sativa* chitin-inducible gibberellin-responsive protein 2 (CIGR2), the *A. thaliana* PAT1 genes (Bolle et al., 2000; Day et al., 2004) and several other genes (Table 1).

OG-PAT-2 includes the *O. sativa* CIGR1 gene (Day et al., 2004) and members from all the analyzed species except *A. thaliana*. BLASTp analyses indicate that Brassicaceae lacks a member of this OG. However, members of OG-PAT-2 were found in *Tarenaya hassleriana* (LOC104798783 and LOC104817890) belonging to the sister group of the Cleomaceae, which indicates that OG-PAT-2 is represented in Brassicales and was probably lost only in the Brassicaceae family. OG-PAT-3 includes the *A. thaliana* gene SCL1 (AT1G21450) and at least one member for each other analyzed species; in monocots, two introns are present in the 3′ coding region, whereas in dicots the gene has the typical mono-exonic structure. OG-PAT-4 includes the *A. thaliana* gene SCL8 (AT5G52510) and at least one member for each other analyzed species with the exception of Amborella.

One or two conserved motifs were detected for each one of these OGs and one region was detected when all the protein sequences of this subfamily were analyzed. A motif conserved among almost all the members of the PAT subfamily was partially overlapping with the motifs detected in OG-specific analysis (Table 2).

### OG-SCL3

In *A. thaliana*, SCL3 was shown promoting gibberellin signaling (by antagonizing master growth repressor DELLA) (Zhang et al., 2011) and was associated with the nuclear hexokinase1 complex (Cho et al., 2006). In *O. sativa* the nine members of OG-SCL3 are the result of both tandem and segmental duplications. A 29aa motif was detected in most of the sequences of this OG, the exceptions all belonging to *O. sativa* (Table 2).

### Dwarf and low-tillering (OG-DLT)

This newly identified OG-DLT was named from the rice member of this family, which is involved in brassinosteroid signaling and its alteration induces dwarf and low-tillering phenotype in rice (Tong et al., 2009). A 15aa conserved motif was detected in all the sequences (Table 2).

### Reduced arbuscular mycorrhization 1 (OG-RAM1)

This newly identified OG was named from the RAM1 gene, involved in the mycorrhizal signaling in *Medicago truncatula* (Gobbato et al., 2012) (Supplementary Data 3). One member of OG-RAM1 was found in each of the analyzed species except *A. thaliana* (missing in all Brassicales). A 21aa-long conserved motif was found in all sequences of this OG, except the *A. trichopoda* one (Table 2).

### Required for arbuscule development 1 (OG-RAD1) subfamily

This newly identified OG-RAD1-1 was named after the RAD1 gene, involved in mycorrhization of *Lotus japonicus* (Xue et al., 2015) (Supplementary Data 3). Members of this OG were found in all the analyzed species except *A. thaliana* (missing in all Brassicales). OG-RAD1-2 members were found only in *A. trichopoda, P. dactylifera, V. vinifera*, and *T. cacao* (Figure 1), but no functional information is available for any member of this OG. One (41aa) and two (20aa and 28aa) conserved motifs were detected in RAD1-1 and RAD1-2 respectively (Table 2).

### Scarecrow-like (OG-SCLA and B)

In those two new subfamilies, no *A. thaliana* members were found for the Scarecrow-like OGs, nor is any functional information available, and consequently they are distinguished by letters. The OG-SCLA includes members from all analyzed species but does not contain any Brassicales member. A 29aa long conserved domain was detected for this member (Table 2).

Members of *A. thaliana* and *O. sativa* are also missing in OG-SCLB, and no members of this OG were found in the *M. acuminata* genome. On the contrary, this OG experienced independent expansion in *P. dactylifera* and *A. trichopoda*, mainly by tandem duplication (Table 1). A 50aa-long conserved motif was detected in several proteins of this OG (Table 2).

### OG-LISCL

This group was named after the *Lilium longiflorum* gene found to regulate meiosis-associated gene regulation (Morohashi et al., 2003) (Supplementary Data 3). The number of OG-LISCL members found in each species is clearly higher than in any other OG. The expansion of this gene family in each species occurred both by segmental and tandem duplications (blocks of consecutive annotation names in Supplementary Data 2). Phylogenetic analysis performed with members of this OG shows that duplications took place during the monocot and dicot radiations, but also by lineage-specific amplification. Members of this OG appear to be involved in different processes. Os04g50060 (alias OsGRAS23) was shown to be involved in drought-stress response and to regulate the expression of stress-responsive genes (Xu K. et al., 2015); At1g07530 (alias SCL14, alias AtGRAS2) is required for the activation of stress-inducible promoters (Fode et al., 2008). Another *O. sativa* gene OsAM18 (Os03g40080; in our study the sequence was considered a pseudogene due to large sequence rearrangements, but close to the OG-LISCL sequences), was found to be involved in mycorrhiza development (Fiorilli et al., 2015).

## Discussion

In order to transfer genetic knowledge from extensively studied model species to important crops with non-optimal features to perform effective experimentation (non-model), it is critical to determine the correct orthology relationship between genes of model and non-model species. Even if the orthology is not a guarantee of function conservation (especially in multi-copy gene families), orthologous genes are still the best candidates for functional annotation transfer. An example of function conservation inside a GRAS is provided by three characterized genes in the LS orthogroup that are all involved in the formation of lateral shoots during vegetative development in phylogenetically distant angiosperm species [*S. lycopersicum* (dicot asterid), *A. thaliana* (dicot rosids) and *O. sativa* (monocot)]. This study aimed at defining the orthology relationships among the GRAS members in angiosperms.

### Orthogroups and phylogeny of the GRAS family

Twenty-nine OGs were recognized in the GRAS family but several groups lacked members in one or more species. The BLASTp analysis on plant protein databases highlighted the absence of representatives of a given OG common to phylogenetically close species, suggesting that the loss predated the origin of some families or orders (Table 1). In particular *A. thaliana*, with a lower number of GRAS members (33), was missing members of 12 OGs as highlighted for other species (Lu et al., 2015; Xu et al., 2016). BLASTp failed to find members of 11 of these OGs in species belonging to the Brassicales order and the remaining one (OG-PAT-2) was missing only in the Brassicaceae family. To a lesser extent, four OGs appeared to have no members in *O. sativa* or in the Poales order. The loss of representative genes of several OGs is striking in contrast with the results of a similar study on another transcription factor family (Cenci et al., 2014). Indeed, among the 40 OGs comprising the NAC gene family (Cenci et al., 2014), 39 had at least one representative in the four species analyzed in the study (i.e., *A. thaliana, V. vinifera, M. acuminata*, and *O. sativa*) and only one was missing in monocots.

Previous genome-wide studies of the GRAS transcription factors involved mainly members of only one or a few species (Tian et al., 2004; Lee et al., 2008; Abarca et al., 2014; Liu and Widmer, 2014; Song et al., 2014; Huang et al., 2015; Lu et al., 2015; Sun et al., 2016) and model species such as *A. thaliana* and *O. sativa* were often used as the reference. These two species have shown evidences of higher evolution rates than observed for *V. vinifera* (Yue et al., 2010) and *M. acuminata*, respectively (D'Hont et al., 2012; Cenci et al., 2013, 2014). Consistent with these observations, in most of the OG phylogenetic trees in this study, *A. thaliana* and *O. sativa* sequences showed longer branch lengths than other dicots and monocots, respectively. A higher evolution rate implies a greater divergence between sequences (more amino acid substitution) which makes the sequence alignment more challenging and increases the background noise that can affect phylogenetic signal. Consequently the *A. thaliana* and *O. sativa* genes, although considered as model plants, are not the optimal sequences to reconstruct difficult gene phylogenies in angiosperms. In order to establish the phylogenetic relationships among GRAS OGs, phylogenetic trees were built with the complete set of 397 sequences and with a subset of 142 sequences belonging to only three species (i.e., *A. trichopoda, V. vinifera*, and *P. dactylifera*). These three species were selected based on three criteria: (i) representation of main groups (basal angiosperm, dicots and monocots, respectively); (ii) representation of all OGs (with two exceptions: OG-NSP2-3 lacks members in *P. dactylifera* and in all monocots and OG-PAT4 lacks members of *A. trichopoda*); (iii) observed slower evolution rate. Overall, the phylogenetic results are consistent with the established OGs as well as with the organization of some OGs in subfamilies (Figures 2, 3). In the eight-species tree a higher number of inconsistencies with the OGs were observed. The inconsistencies could be due to the limited number of aligned positions retained for the analysis (151 and 180 in the eight-species and three-species trees, respectively). The phylogenetic consistencies of OG grouped in subfamilies was confirmed by conducting phylogenetic analyses on subsets of sequences belonging to these subfamilies (that are based on longer sequence alignments): clustering completely consistent with OG definition and high branch support (aLRT always higher than 0.9) were observed (Supplementary Data 2; Supplementary Figures 1–38).

In the phylogenetic tree (Figure 3), the Amborella sequences that are not included in any OG (i.e., LOC18433705 and LOC18433702) are clustered with the NSP2 subfamily, as sister group of OG-NSP2-3. These two sequences form a very well supported cluster (aLRT = 0.999). The most likely scenario is the loss of the orthologous gene in the ancestor of the other angiosperms, although a duplication from the *A. trichopoda* sequence included in OG-NSP2-3 followed by extensive diversification cannot be ruled out.

Concerning the deep nodes, i.e., the relationships among subfamilies and isolated OGs, the phylogenetic signal is less clear. Strong support was observed for the cluster, including SHR, SCL32, and NSP1 subfamilies (aLRT = 0.999), as already observed in previous studies (Tian et al., 2004; Lee et al., 2008; Wu et al., 2014) and for the clusters including also PAT subfamily (aLRT = 0.977). The remaining basal nodes have branch support lower than 0.9. According to Zhang et al. (2012), who rooted the phylogenetic tree of the GRAS gene family in *A. thaliana* with the bacterial GRAS genes, the first duplication separated the LISCL ancestor from the ancestor of all other GRAS families. However, the *A. thaliana*-specific lineage lost several GRAS members that could explain this result. In particular, the SCLB subfamily which is missing in *A. thaliana*, was clustered with OG-LISCL in our phylogenetic analysis (Figure 2).

The presence of an *A. trichopoda* representative close to almost all OGs is consistent with an ancient diversification of the different clades, as shown in a phylogenetic analysis that includes basal Viridiplantae (Engstrom, 2011). The unique exception is OG-PAT-4, for which no close *A. trichopoda* gene was found. The most likely hypothesis is that *A. trichopoda* lost the gene corresponding to OG-PAT-4. In fact, the closest *A. trichopoda* gene to OG-PAT-4, i.e., LOC18432892, is included in the well supported OG-PAT-3 cluster (Supplementary Data 2; Supplementary Figure 29) and it is unlikely that it derived from the common ancestors OG-PAT-3 and -4.

At least 29 ancestor genes were present in the angiosperm lineage before the Amborella split. Since all OGs based on the monocot-dicot split contain one or more *A. trichopoda* sequences, we can conclude that no GRAS gene duplication took place between the basal angiosperm Amborella split from the other angiosperms and the monocot-dicot split.

This phylogenetic tree (Figure 2) is based on species with the most complete GRAS OG set and with extensive taxonomic representation. A detailed comparison of phylogenetic results with the ones obtained in previous study (that mainly focused on *A. thaliana* and *O. sativa)* has been complicated by the detected loss of GRAS members of these species in some OGs (12 and 4, respectively). The main clusters of sequences described in previous studies were consistently found in our analysis. In addition, the wide representation of angiosperm species allowed us to detect and describe five new and well differentiated subfamilies: DLT, RAD1 (including 2 OGs), RAM1, SCLA, and SCLB, the last two being not yet functionally characterized. The existence of new subfamilies was already partially detected by Grimplet et al. (2016) in their analysis of *V. vinifera* GRAS: GRAS8 group corresponds to OG-DLT and OG-SCLA, GRASV1 to RAD1 subfamily and OG-RAM1, and GRASV2 to SCLB. However, our analysis allowed us to classify with higher precision the suggested *V. vinifera* subfamilies (e.g., GRASV1 contains genes belonging to OG-RAD1-1, OG-RAD1-2, and RAM1). The variable number of groups or subfamilies that are defined according the different studies reflects both the presence/absence of GRAS members and the arbitrary choices of the authors. In our study, we grouped the OGs into 17 subfamilies (of which eight include two or more OGs) based on arbitrary thresholds of sequence similarity. However, these subfamilies could be decreased or increased in number if, for example, we decided to join the closer clusters NSP2 and HAM or to separate the cluster containing the OG-PAT-3 and -4 genes from the other PAT OGs (Figure 2). Conversely, the OGs are not based on sequence similarity, but they reflect the established phylogenetic history, as all the genes included in an OG derived from a common ancestor existing before the split between monocots and dicots. Obviously, one could base the orthology study on a different point of the phylogenetic history (e.g., the split between asterids and rosids) and obtain different OG numbers, even if using the same objective analysis. In our study, we focused on the monocot/dicot split that includes almost all the food crops to facilitate establishing the orthology relationships between models and plants of agronomic interest. In addition, unbiased comparisons could be performed with a similar study on NAC transcription factors, where the orthology definition was also based on the monocot/dicot split.

### Conserved motifs

The GRAS family is characterized by the presence of the GRAS domain in the C-terminal sequence part which is composed of five subdomains. The MEME analysis performed on a set of sequences representative of all OGs confirmed the high level of conservation of the GRAS domain.

The MEME analysis in each OG allowed identifying, in addition to the GRAS domain, one or two conserved motifs in the *N*-terminal portion of several OGs (Table 2). These motifs appear mainly OG-specific, and the analysis of subfamilies (grouping 2–4 OGs) failed to detect shared conserved motifs, with the exception of the DELLA domain in the OG-DELLA-1 and -2 and the conserved motifs in all 4 OGs included in the PAT family.

The detected conserved motifs lie in a highly variable region, classed as an intrinsically disordered sequence (Sun et al., 2011, 2012). The conservation in orthologous genes of species spanning the angiosperm radiation implies that a selective pressure was exercised on these sequence parts and suggests their involvement in protein functionality.

### Orthogroups evolution history and γ WGD of dicots

The evolutionary history of the GRAS family appears complex. Most of the duplications and diversification took place before the angiosperm radiation. Additional duplications could be observed during the evolution of angiosperm lineages. In particular, in 8 of 29 OGs (DELLA-1, HAM-II, LISCL, PAT-1, PAT-3, SCL3-1, SCL3-2, and SCLB), dicot-specific duplications or triplications were clearly observed. A WGD event (γ hexaploidization) pre-dated the radiation of all dicot species considered in this study (Figure 1; Jaillon et al., 2007; Cenci et al., 2010). Since Sun et al. (2016) identified some GRAS genes associated with γ WGD-duplicated blocks in the *V. vinifera* genome, we tried to verify if the observed dicot-specific duplications can be related to the γ WGD.

The OG-DELLA-1 tree shows two eudicot clusters, each one containing at least one gene of each studied dicots species (Supplementary Figure 22). The *V. vinifera* genes are located in chromosomes 1 and 14, which contain, along with the chromosome 17, large duplicated segments. However, these genes are not included in any known duplicated segments. The clusters also include two *C. canephora* genes (Cc11_g08290 and Cc07_g13590). As for *V. vinifera*, chromosomes 11, 7, and 4 share large triplicated segments that do not include the GRAS gene. A closer examination of the surrounding genomic regions of the GRAS genes reveals the presence of a small duplicated segment, which includes the GRAS genes and 10 other gene pairs (Supplementary Table 2). This segment duplication was not detected in former analyses, likely due to its short size and a post-duplication amplification of genes in the chromosome 11 segment. Since the GRAS genes lie in an extension of regions assigned to γ WGD, their duplication can be considered associated with this event.

In the OG-HAM-II tree, two clusters contain genes from *C. canephora, V. vinifera*, and *T. cacao* (Supplementary Figure 20). In *V. vinifera*, the GRAS genes are located at the end of a duplicated segment in Chromosomes 2 and 15 (Jaillon et al., 2007). *C. canephora* genes are located in a duplicated segment containing 37 genes (Denoeud et al., 2014). Both the duplicated segments were associated with γ WGD.

The LISCL orthogroup contains a very large number of members originated by both segmental and tandem duplication in all studied angiosperm species, whereas only one sequence was found in *A. trichopoda* for this OG. In the dicot phylogenetic cluster (Supplementary Figure 38), three well-supported clusters can be observed: one contains only four genes, one member for each dicot species; a second cluster does not contain any *A. thaliana* members and a species-specific tandem amplification took place in chromosome 13 of *V. vinifera*; the third cluster is the largest one and contains additional segmental and tandem duplications involving all the studied dicots. In each one of the three above-described clusters a *V. vinifera* gene is included, assigned to a triplicated segment associated with the γ WGD (Sun et al., 2016).

The dicot genes in OG-PAT-1 phylogenetic tree (Supplementary Figure 25) are organized in three clusters. Two *V. vinifera* and two *T. cacao* genes were found in duplication associated with the γ WGD (Argout et al., 2011; Sun et al., 2016); in both cases, the genes are included in the closer dicot specific clusters. For the third dicot cluster (the basal one), it is not possible to establish relationships with duplicated segments due to GRAS genes unassigned to *V. vinifera* or *C. canephora* chromosomes [LOC100245041 (*V. vinifera*) and Cc00_g06820 (*C. canephora*)] and the *T. cacao* gene TCM_030393 not included in duplication segments). Consequently the third block cannot be shown to originate from the γ WGD.

In the OG-PAT-3 tree only a dicot cluster is present, but one of the two *V. vinifera* genes (LOC100854518) has an unexpected position, being basal for the complete cluster (Supplementary Figure 27). Since the *V. vinifera* genes were found associated with the γ WGD (Sun et al., 2016), it can be concluded that one of duplicated copies was retained in *V. vinifera*, whereas it was lost in all other analyzed dicots.

The OG-SCL3 tree includes three dicot clusters (Supplementary Figure 30); one of them includes only two GRAS sequences from *T. cacao* and *V. vinifera* and has long branches. The unexpected position of this cluster inside the *O. sativa* specific cluster (Supplementary Figure 30) could be an artifact due to the LBA phenomenon. The other two dicot clusters, each containing sequences from *T. cacao, V. vinifera* and *C. canephora*, have expected positions in a well-supported dicot specific cluster and could result from the γ WGD. However, none of these genes could be associated with duplicated segments in *V. vinifera, T. cacao*, and *C. canephora*.

In the OG-RAD1-1 tree, the dicot cluster is divided in two sub-clusters each containing a gene from *V. vinifera, T. cacao*, and *C. canephora* (Supplementary Figure 33). The *C. canephora* genes lie in duplicated segments containing 38 duplicated genes and the *V. vinifera* genes were found in duplicated segments associated with the γ WGD (Sun et al., 2016).

The dicot cluster of the OG-SCLB tree appears divided in three sub-clusters, even though one has low support (aLRT = 0.67) (Supplementary Figure 37). Each dicot sub-cluster has at least one member of *C. canephora* and *T. cacao*. Two of these dicot sub-clusters appear to be the result of a tandem duplication taking place before the rosid/asterid split. In fact, the genes of these dicot sub-clusters are arranged in tandem in both *T. cacao* (TCM_021350 and TCM_021351) and *C. canephora* [Cc03-g00940-Cc03-g00950 and the unannotated Cc03:(712804.714285)]. The *T. cacao* and *C. canephora* genes in the third dicot sub-cluster lie in different chromosomes (Cc01_g07010 and TCM-04818) which excludes their origin from tandem duplication, and are thus not included in known duplicated segments. However, Sun et al. (2016) found the unique *V. vinifera* gene (in the OG-SCLB tree in segment of chromosome 7) duplicated in chromosome 5, where there is another *V. vinifera* gene that was considered as a pseudogene in this study. When this sequence was considered in the phylogenetic analysis (data not shown), it was included in the largest dicot sub-cluster, suggesting the γ WGD was involved in the dicot amplification of the OG-SCLB.

In conclusion, for at least seven of the eight OGs for which dicot specific duplications were observed, the γ WGD appears to be involved, and in the remaining OG, the γ WGD cannot be ruled out. Moreover, segmental duplications and tandem duplications appear to participate in the gene number amplification as previously reported (Tian et al., 2004; Grimplet et al., 2016), especially in the OG-LISCL, which includes a dramatically higher number of GRAS genes.

### GRAS family evolution in angiosperm

When compared with the evolution of the NAC TF family, the expansion of the GRAS gene family appears limited only to a few OGs, whereas the number of GRAS members was constant in the majority of its OGs. The preferential retention of copies during the whole genome duplication and following fragmentation observed for other transcription factors (Maere et al., 2005) seems to be less extensive in the GRAS gene family. An average of 50 GRAS members belonging to 29 OGs were counted in the eight species studied here, which strongly contrasts with the findings in NAC TFs where we defined 40 OGs for 100 NAC members were counted in the analyzed species (Cenci et al., 2014). If the number of annotated members of NAC and GRAS transcription factor is compared (Supplementary Figures 39, 40), it can be highlighted that the NAC gene family experienced an expansion in the higher plant lineages, as their member number is clearly higher than in *P. patens* and *S. moellendorffii*, a moss and a lycophyte, respectively. By contrast, in GRAS TFs the number of GRAS genes in these species is comparable.

Moreover, the complete loss of OG members in a given species or lineage was not observed in a similar study performed on the NAC TFs (Cenci et al., 2014). It is worth underlining that most of the OGs that are missing members of one or more species belong to subfamilies that include more than one OG. That these OGs lack some lineages suggests that, in spite of their ancient differentiation, GRAS family members of close OGs maintained some degree of redundancy and the complete loss of OG representatives in a given lineage seems to be compensated by the presence of members of close OGs. These observations are consistent with the results of Lee et al. (2008) who reported normal phenotype for a panel of 23 loss-of-function mutants of different functionally uncharacterized *A. thaliana* GRAS genes. Partial redundancy was also shown in *A. thaliana* for four HAM genes belonging to the two close OG-HAM-I and -II (Engstrom et al., 2011).

The analysis of GRAS TF in additional species' genomes, of which availability is increasing, might allow improving the global panorama of the GRAS gene family in angiosperms. However, since the panel of species used in this study was designed to provide a wide view of the angiosperms, the discovery of new GRAS OGs is highly unlikely. On the contrary, data from additional species will allow a more precise reconstruction of the history of duplication and loss of members in each OG. At the same time, a precise GRAS classification will help to better characterize the evolution of specific members (e.g., two *O. sativa* member of OG-DELLA-2 have lost their DELLA domain).

Finally, the GRAS classification in OG is also expected to be a useful tool in studies that are not exclusively devoted to the GRAS family. As an example, a classification was performed on 18 GRAS genes found in a panel of 45 transcription factors up-regulated in *Lotus japonicus* roots with mycorrhizal colonization (Xue et al., 2015). The classification of these genes identified the OGs mainly responsive to the mycorrhiza development, i.e., the OG-SCL3 (4 genes), the OG-SCLB (3 genes), and that the RAD1 subfamily has the most of the up-regulated genes (4 genes). It also highlighted several OGs known to be involved in root development (SHR and SCR) in interaction with soil microorganisms involved in nodulation (NSP2) and arbuscular mycorrhiza formation (RAD1 and RAM1) (Supplementary Table 3). Therefore, this study provides a solid framework of the orthology relationships in the angiosperm GRAS transcription factors, thus increasing the accuracy of ortholog identification in model species and facilitating the identification of agronomically important genes related to various traits such abiotic stress tolerance.

## Author contributions

MR and AC conceived and designed the experiments. AC conducted genome evolution analyses including gene family and phylogenetic analysis. MR and AC conducted protein domain analyses. MR and AC wrote the manuscript.

## Funding

This work was supported by CGIAR Fund Donors and CGIAR Research Programme on Roots, Tubers and Bananas.

### Conflict of interest statement

The authors declare that the research was conducted in the absence of any commercial or financial relationships that could be construed as a potential conflict of interest.

## Supplementary material

Supplementary Data 1 **Multi-fasta file the 397 GRAS protein sequences**.

Supplementary Data 2 **Unrooted trees obtained in this study (Newick format)**. The two first trees corresponds to Figures 2, 3; OG-XX-N indicate the 29 unrooted trees corresponding to each OG; XX subfamily indicate the 8 unrooted trees corresponding to the subfamily including more than one OG.

Supplementary Data 3 **Multi-fasta of published sequenced of functionally characterized GRAS protein genes not included in the 8 species**.

Supplementary Table 1 **Gene list of the eight studied species according to the OG assignation**. For each gene, names in different annotations and nomenclature used in previously published study are provided.

Supplementary Table 2 **Genes in duplicated segments on *C. canephora* chromosomes 7 and 11 including two DELLA-1 genes**.

Supplementary Table 3 **Classification of GRAS genes up-regulated in *L. japonicus* roots with mycorrhizal colonization (Xue et al., 2015)**.

Supplementary Figures 1–40 **Individual phylogenies for orthogroups and subfamilies**.

## References

[B1] Abarca D. Pizarro A. Hernández I. Sánchez C. Solana S. P. Del Amo A.. (2014). The GRAS gene family in pine: transcript expression patterns associated with the maturation-related decline of competence to form adventitious roots. BMC Plant Biol. 14:354. 10.1186/s12870-014-0354-8 25547982 PMC4302573

[B2] Albert V. A. Barbazuk W. B. de Pamphilis C. W. Der J. P. Leebens-Mack J. Ma H. (2013). The Amborella genome and the evolution of flowering plants. Science 342:1241089 10.1126/science.1241089 24357323

[B3] Al-Mssallem I. S. Hu S. Zhang X. Lin Q. Liu W. Tan J.. (2013). Genome sequence of the date palm *Phoenix dactylifera* L. Nat. Commun. 4:2274. 10.1038/ncomms3274 23917264 PMC3741641

[B4] Altenhoff A. M. Gil M. Gonnet G. H. Dessimoz C. (2013). Inferring hierarchical orthologous groups from orthologous gene pairs. PLoS ONE 8:e53786. 10.1371/journal.pone.0053786 23342000 PMC3544860

[B5] Argout X. Salse J. Aury J.-M. Guiltinan M. J. Droc G. Gouzy J.. (2011). The genome of *Theobroma cacao*. Nat. Genet. 43, 101–108. 10.1038/ng.736 21186351

[B6] Bailey T. L. Johnson J. Grant C. E. Noble W. S. (2015). The MEME suite. Nucleic Acids Res. 43, W39–W49. 10.1093/nar/gkv416 25953851 PMC4489269

[B7] Bolle C. (2004). The role of GRAS proteins in plant signal transduction and development. Planta 218, 683–692. 10.1007/s00425-004-1203-z 14760535

[B8] Bolle C. Koncz C. Chua N. H. (2000). PAT1, a new member of the GRAS family, is involved in phytochrome A signal transduction. Genes Dev. 14, 1269–1278. 10.1101/gad.14.10.1269 10817761 PMC316623

[B9] Castresana J. (2000). Selection of conserved blocks from multiple alignments for their use in phylogenetic analysis. Mol. Biol. Evol. 17, 540–552. 10.1093/oxfordjournals.molbev.a026334 10742046

[B10] Cenci A. Combes M.-C. Lashermes P. (2010). Comparative sequence analyses indicate that Coffea (Asterids) and Vitis (Rosids) derive from the same paleo-hexaploid ancestral genome. Mol. Genet. Genomics 283, 493–501. 10.1007/s00438-010-0534-7 20361338

[B11] Cenci A. Combes M. C. Lashermes P. (2013). Differences in evolution rates among eudicotyledon species observed by analysis of protein divergence. J. Hered. 104, 459–464. 10.1093/jhered/est025 23596284

[B12] Cenci A. Guignon V. Roux N. Rouard M. (2014). Genomic analysis of NAC transcription factors in banana (*Musa acuminata*) and definition of NAC orthologous groups for monocots and dicots. Plant Mol. Biol. 85, 63–80. 10.1007/s11103-013-0169-2 24570169 PMC4151281

[B13] Cho Y.-H. Yoo S.-D. Sheen J. (2006). Regulatory functions of nuclear hexokinase1 complex in glucose signaling. Cell 127, 579–589. 10.1016/j.cell.2006.09.028 17081979

[B14] Conte M. G. Gaillard S. Lanau N. Rouard M. Périn C. (2008). GreenPhylDB: a database for plant comparative genomics. Nucleic Acids Res. 36, D991–D998. 10.1093/nar/gkm934 17986457 PMC2238940

[B15] Cui H. Kong D. Liu X. Hao Y. (2014). SCARECROW, SCR-LIKE 23 and SHORT-ROOT control bundle sheath cell fate and function in *Arabidopsis thaliana*. Plant J. Cell Mol. Biol. 78, 319–327. 10.1111/tpj.12470 24517883

[B16] Day R. B. Tanabe S. Koshioka M. Mitsui T. Itoh H. Ueguchi-Tanaka M.. (2004). Two rice GRAS family genes responsive to *N*-acetylchitooligosaccharide elicitor are induced by phytoactive gibberellins: evidence for cross-talk between elicitor and gibberellin signaling in rice cells. Plant Mol. Biol. 54, 261–272. 10.1023/B:PLAN.0000028792.72343.ee 15159627

[B17] Denoeud F. Carretero-Paulet L. Dereeper A. Droc G. Guyot R. Pietrella M.. (2014). The coffee genome provides insight into the convergent evolution of caffeine biosynthesis. Science 345, 1181–1184. 10.1126/science.1255274 25190796

[B18] Dereeper A. Bocs S. Rouard M. Guignon V. Ravel S. Tranchant-Dubreuil C.. (2015). The coffee genome hub: a resource for coffee genomes. Nucleic Acids Res. 43, D1028–D1035. 10.1093/nar/gku1108 25392413 PMC4383925

[B19] Dereeper A. Guignon V. Blanc G. Audic S. Buffet S. Chevenet F.. (2008). Phylogeny.fr: robust phylogenetic analysis for the non-specialist. Nucleic Acids Res. 36, W465–W469. 10.1093/nar/gkn180 18424797 PMC2447785

[B20] D'Hont A. Denoeud F. Aury J.-M. Baurens F.-C. Carreel F. Garsmeur O.. (2012). The banana (*Musa acuminata*) genome and the evolution of monocotyledonous plants. Nature 488, 213–217. 10.1038/nature11241 22801500

[B21] Di Laurenzio L. Wysocka-Diller J. Malamy J. E. Pysh L. Helariutta Y. Freshour G.. (1996). The SCARECROW gene regulates an asymmetric cell division that is essential for generating the radial organization of the Arabidopsis root. Cell 86, 423–433. 10.1016/S0092-8674(00)80115-4 8756724

[B22] Engstrom E. M. (2011). Phylogenetic analysis of GRAS proteins from moss, lycophyte and vascular plant lineages reveals that GRAS genes arose and underwent substantial diversification in the ancestral lineage common to bryophytes and vascular plants. Plant Signal. Behav. 6, 850–854. 10.4161/psb.6.6.15203 21543899 PMC3218485

[B23] Engstrom E. M. Andersen C. M. Gumulak-Smith J. Hu J. Orlova E. Sozzani R.. (2011). Arabidopsis homologs of the petunia hairy meristem gene are required for maintenance of shoot and root indeterminacy. Plant Physiol. 155, 735–750. 10.1104/pp.110.168757 21173022 PMC3032463

[B24] Fiorilli V. Volpe V. Zanini S. Vallino M. Abbà S. Bonfante P. (2015). A rice GRAS gene has an impact on the success of Arbuscular mycorrhizal colonization. Am. J. Plant Sci. 6:1905 10.4236/ajps.2015.612191

[B25] Fode B. Siemsen T. Thurow C. Weigel R. Gatz C. (2008). The Arabidopsis GRAS protein SCL14 interacts with class II TGA transcription factors and is essential for the activation of stress-inducible promoters. Plant Cell 20, 3122–3135. 10.1105/tpc.108.058974 18984675 PMC2613660

[B26] Gobbato E. Marsh J. F. Vernié T. Wang E. Maillet F. Kim J.. (2012). A GRAS-type transcription factor with a specific function in mycorrhizal signaling. Curr. Biol. 22, 2236–2241. 10.1016/j.cub.2012.09.044 23122845

[B27] Greb T. Clarenz O. Schafer E. Muller D. Herrero R. Schmitz G.. (2003). Molecular analysis of the LATERAL SUPPRESSOR gene in Arabidopsis reveals a conserved control mechanism for axillary meristem formation. Genes Dev. 17, 1175–1187. 10.1101/gad.260703 12730136 PMC196050

[B28] Grimplet J. Agudelo-Romero P. Teixeira R. T. Martinez-Zapater J. M. Fortes A. M. (2016). Structural and functional analysis of the GRAS gene family in grapevine indicates a role of GRAS proteins in the control of development and stress responses. Front. Plant Sci. 7:353. 10.3389/fpls.2016.00353 27065316 PMC4811876

[B29] Guindon S. Delsuc F. Dufayard J.-F. Gascuel O. (2009). Estimating maximum likelihood phylogenies with PhyML. Methods Mol. Biol. 537, 113–137. 10.1007/978-1-59745-251-9_6 19378142

[B30] Heidstra R. Welch D. Scheres B. (2004). Mosaic analyses using marked activation and deletion clones dissect Arabidopsis SCARECROW action in asymmetric cell division. Genes Dev. 18, 1964–1969. 10.1101/gad.305504 15314023 PMC514176

[B31] Hirsch S. Oldroyd G. E. D. (2009). GRAS-domain transcription factors that regulate plant development. Plant Signal. Behav. 4, 698–700. 10.4161/psb.4.8.9176 19820314 PMC2801379

[B32] Huang W. Xian Z. Kang X. Tang N. Li Z. (2015). Genome-wide identification, phylogeny and expression analysis of GRAS gene family in tomato. BMC Plant Biol. 15:209. 10.1186/s12870-015-0590-6 26302743 PMC4549011

[B33] Ikeda A. Ueguchi-Tanaka M. Sonoda Y. Kitano H. Koshioka M. Futsuhara Y.. (2001). slender rice, a constitutive gibberellin response mutant, is caused by a null mutation of the SLR1 gene, an ortholog of the height-regulating gene GAI/RGA/RHT/D8. Plant Cell 13, 999–1010. 10.1105/tpc.13.5.999 11340177 PMC135552

[B34] Itoh H. Shimada A. Ueguchi-Tanaka M. Kamiya N. Hasegawa Y. Ashikari M.. (2005). Overexpression of a GRAS protein lacking the DELLA domain confers altered gibberellin responses in rice. Plant J. 44, 669–679. 10.1111/j.1365-313X.2005.02562.x 16262715

[B35] Jaillon O. Aury J.-M. Noel B. Policriti A. Clepet C. Casagrande A.. (2007). The grapevine genome sequence suggests ancestral hexaploidization in major angiosperm phyla. Nature 449, 463–467. 10.1038/nature06148 17721507

[B36] Katoh K. Standley D. M. (2013). MAFFT multiple sequence alignment software version 7: improvements in performance and usability. Mol. Biol. Evol. 30, 772–780. 10.1093/molbev/mst010 23329690 PMC3603318

[B37] Kaul S. Koo H. Jenkins J. Rizzo M. Rooney T. Tallon L. (2000). Analysis of the genome sequence of the flowering plant *Arabidopsis thaliana*. Nature 408, 796–815. 10.1038/35048692 11130711

[B38] Kuzniar A. van Ham R. C. H. J. Pongor S. Leunissen J. A. M. (2008). The quest for orthologs: finding the corresponding gene across genomes. Trends Genet. 24, 539–551. 10.1016/j.tig.2008.08.009 18819722

[B39] Lee M.-H. Kim B. Song S.-K. Heo J.-O. Yu N.-I. Lee S. A.. (2008). Large-scale analysis of the GRAS gene family in *Arabidopsis thaliana*. Plant Mol. Biol. 67, 659–670. 10.1007/s11103-008-9345-1 18500650

[B40] Letunic I. Bork P. (2016). Interactive tree of life (iTOL) v3: an online tool for the display and annotation of phylogenetic and other trees. Nucleic Acids Res. 44, W242–W245. 10.1093/nar/gkw290 27095192 PMC4987883

[B41] Li W. Cowley A. Uludag M. Gur T. McWilliam H. Squizzato S.. (2015). The EMBL-EBI bioinformatics web and programmatic tools framework. Nucleic Acids Res. 43, W580–W584. 10.1093/nar/gkv279 25845596 PMC4489272

[B42] Li X. Qian Q. Fu Z. Wang Y. Xiong G. Zeng D.. (2003). Control of tillering in rice. Nature 422, 618–621. 10.1038/nature01518 12687001

[B43] Liu W. Kohlen W. Lillo A. Op den Camp R. Ivanov S. Hartog M.. (2011). Strigolactone biosynthesis in *Medicago truncatula* and rice requires the symbiotic GRAS-type transcription factors NSP1 and NSP2. Plant Cell 23, 3853–3865. 10.1105/tpc.111.089771 22039214 PMC3229154

[B44] Liu X. Widmer A. (2014). Genome-wide comparative analysis of the GRAS gene family in populus, Arabidopsis and rice. Plant Mol. Biol. Rep. 32, 1129–1145. 10.1007/s11105-014-0721-5

[B45] Lu J. Wang T. Xu Z. Sun L. Zhang Q. (2015). Genome-wide analysis of the GRAS gene family in *Prunus mume*. Mol. Genet. Genomics 290, 303–317. 10.1007/s00438-014-0918-1 25245166

[B46] Lyons E. Pedersen B. Kane J. Freeling M. (2008). The value of nonmodel genomes and an example using SynMap within CoGe to dissect the hexaploidy that predates the rosids. Trop. Plant Biol. 1, 181–190. 10.1007/s12042-008-9017-y

[B47] Ma H.-S. Liang D. Shuai P. Xia X.-L. Yin W.-L. (2010). The salt- and drought-inducible poplar GRAS protein SCL7 confers salt and drought tolerance in *Arabidopsis thaliana*. J. Exp. Bot. 61, 4011–4019. 10.1093/jxb/erq217 20616154 PMC2935874

[B48] Maere S. De Bodt S. Raes J. Casneuf T. Van Montagu M. Kuiper M.. (2005). Modeling gene and genome duplications in eukaryotes. Proc. Natl. Acad. Sci. U.S.A. 102, 5454–5459. 10.1073/pnas.0501102102 15800040 PMC556253

[B49] Morohashi K. Minami M. Takase H. Hotta Y. Hiratsuka K. (2003). Isolation and characterization of a novel GRAS gene that regulates meiosis-associated gene expression. J. Biol. Chem. 278, 20865–20873. 10.1074/jbc.M301712200 12657631

[B50] Park J. Nguyen K. T. Park E. Jeon J.-S. Choi G. (2013). DELLA proteins and their interacting RING Finger proteins repress gibberellin responses by binding to the promoters of a subset of gibberellin-responsive genes in Arabidopsis. Plant Cell 25, 927–943. 10.1105/tpc.112.108951 23482857 PMC3634697

[B51] Pysh L. D. Wysocka-Diller J. W. Camilleri C. Bouchez D. Benfey P. N. (1999). The GRAS gene family in Arabidopsis: sequence characterization and basic expression analysis of the SCARECROW-LIKE genes. Plant J. Cell Mol. Biol. 18, 111–119. 10.1046/j.1365-313X.1999.00431.x 10341448

[B52] Rouard M. Guignon V. Aluome C. Laporte M.-A. Droc G. Walde C.. (2011). GreenPhylDB v2.0: comparative and functional genomics in plants. Nucleic Acids Res. 39, D1095–D1102. 10.1093/nar/gkq811 20864446 PMC3013755

[B53] Schumacher K. Schmitt T. Rossberg M. Schmitz G. Theres K. (1999). The Lateral suppressor (Ls) gene of tomato encodes a new member of the VHIID protein family. Proc. Natl. Acad. Sci. U.S.A. 96, 290–295. 10.1073/pnas.96.1.290 9874811 PMC15132

[B54] Sequencing Project International Rice Genome (2005). The map-based sequence of the rice genome. Nature 436, 793–800. 10.1038/nature03895 16100779

[B55] Silverstone A. L. Ciampaglio C. N. Sun T. (1998). The Arabidopsis RGA gene encodes a transcriptional regulator repressing the gibberellin signal transduction pathway. Plant Cell 10, 155–169. 10.1105/tpc.10.2.155 9490740 PMC143987

[B56] Smit P. Raedts J. Portyanko V. Debellé F. Gough C. Bisseling T.. (2005). NSP1 of the GRAS protein family is essential for rhizobial Nod factor-induced transcription. Science 308, 1789–1791. 10.1126/science.1111025 15961669

[B57] Song X.-M. Liu T.-K. Duan W.-K. Ma Q.-H. Ren J. Wang Z.. (2014). Genome-wide analysis of the GRAS gene family in Chinese cabbage (*Brassica rapa* ssp. *pekinensis*). Genomics 103, 135–146. 10.1016/j.ygeno.2013.12.004 24365788

[B58] Stuurman J. Jäggi F. Kuhlemeier C. (2002). Shoot meristem maintenance is controlled by a GRAS-gene mediated signal from differentiating cells. Genes Dev. 16, 2213–2218. 10.1101/gad.230702 12208843 PMC186665

[B59] Sun X. Jones W. T. Rikkerink E. H. A. (2012). GRAS proteins: the versatile roles of intrinsically disordered proteins in plant signalling. Biochem. J. 442, 1–12. 10.1042/BJ20111766 22280012

[B60] Sun X. Xie Z. Zhang C. Mu Q. Wu W. Wang B.. (2016). A characterization of grapevine of GRAS domain transcription factor gene family. Funct. Integr. Genomics 16, 347–363. 10.1007/s10142-016-0479-y 26842940

[B61] Sun X. Xue B. Jones W. T. Rikkerink E. Dunker A. K. Uversky V. N. (2011). A functionally required unfoldome from the plant kingdom: intrinsically disordered N-terminal domains of GRAS proteins are involved in molecular recognition during plant development. Plant Mol. Biol. 77, 205–223. 10.1007/s11103-011-9803-z 21732203

[B62] Tamura K. Stecher G. Peterson D. Filipski A. Kumar S. (2013). MEGA6: molecular evolutionary genetics analysis version 6.0. Mol. Biol. Evol. 30, 2725–2729. 10.1093/molbev/mst197 24132122 PMC3840312

[B63] Tian C. Wan P. Sun S. Li J. Chen M. (2004). Genome-wide analysis of the GRAS gene family in rice and Arabidopsis. Plant Mol. Biol. 54, 519–532. 10.1023/B:PLAN.0000038256.89809.57 15316287

[B64] Tong H. Jin Y. Liu W. Li F. Fang J. Yin Y.. (2009). DWARF AND LOW-TILLERING, a new member of the GRAS family, plays positive roles in brassinosteroid signaling in rice. Plant J. Cell Mol. Biol. 58, 803–816. 10.1111/j.1365-313X.2009.03825.x 19220793

[B65] Trachana K. Larsson T. A. Powell S. Chen W. Doerks T. Muller J.. (2011). Orthology prediction methods: a quality assessment using curated protein families. Bioessays 33, 769–780. 10.1002/bies.201100062 21853451 PMC3193375

[B66] Wang Y. Shi S. Zhou Y. Zhou Y. Yang J. Tang X. (2016). Genome-wide identification and characterization of GRAS transcription factors in sacred lotus (*Nelumbo nucifera*). PeerJ 4:e2388. 10.7717/peerj.2388 27635351 PMC5012262

[B67] Wu N. Zhu Y. Song W. Li Y. Yan Y. Hu Y. (2014). Unusual tandem expansion and positive selection in subgroups of the plant GRAS transcription factor superfamily. BMC Plant Biol. 14:373. 10.1186/s12870-014-0373-5 25524588 PMC4279901

[B68] Wysocka-Diller J. W. Helariutta Y. Fukaki H. Malamy J. E. Benfey P. N. (2000). Molecular analysis of SCARECROW function reveals a radial patterning mechanism common to root and shoot. Development 127, 595–603. 10631180 10.1242/dev.127.3.595

[B69] Xu F. Xiao W. Li J. Ding C. Li S. Liu W. (2015). NtGRAS-R1, a topping responsive transcription regulator in tobacco roots. Acta Physiol. Plant. 37:191 10.1007/s11738-015-1940-6

[B70] Xu K. Chen S. Li T. Ma X. Liang X. Ding X.. (2015). OsGRAS23, a rice GRAS transcription factor gene, is involved in drought stress response through regulating expression of stress-responsive genes. BMC Plant Biol. 15:141. 10.1186/s12870-015-0532-3 26067440 PMC4465154

[B71] Xu W. Chen Z. Ahmed N. Han B. Cui Q. Liu A. (2016). Genome-wide identification, evolutionary analysis, and stress responses of the GRAS gene family in castor beans. Int. J. Mol. Sci. 17:1004. 10.3390/ijms17071004 27347937 PMC4964380

[B72] Xue L. Cui H. Buer B. Vijayakumar V. Delaux P.-M. Junkermann S.. (2015). Network of GRAS transcription factors involved in the control of arbuscule development in *Lotus japonicus*. Plant Physiol. 167, 854–871. 10.1104/pp.114.255430 25560877 PMC4348782

[B73] Yu N. Luo D. Zhang X. Liu J. Wang W. Jin Y.. (2014). A DELLA protein complex controls the arbuscular mycorrhizal symbiosis in plants. Cell Res. 24, 130–133. 10.1038/cr.2013.167 24343576 PMC3879709

[B74] Yue J.-X. Li J. Wang D. Araki H. Tian D. Yang S. (2010). Genome-wide investigation reveals high evolutionary rates in annual model plants. BMC Plant Biol. 10:242. 10.1186/1471-2229-10-242 21062446 PMC3095324

[B75] Zhang D. Iyer L. M. Aravind L. (2012). Bacterial GRAS domain proteins throw new light on gibberellic acid response mechanisms. Bioinformatics 28, 2407–2411. 10.1093/bioinformatics/bts464 22829623 PMC3463117

[B76] Zhang Z.-L. Ogawa M. Fleet C. M. Zentella R. Hu J. Heo J.-O.. (2011). Scarecrow-like 3 promotes gibberellin signaling by antagonizing master growth repressor DELLA in Arabidopsis. Proc. Natl. Acad. Sci. U.S.A. 108, 2160–2165. 10.1073/pnas.1012232108 21245327 PMC3033277

[B77] Zhao H. Dong L. Sun H. Li L. Lou Y. Wang L.. (2016). Comprehensive analysis of multi-tissue transcriptome data and the genome-wide investigation of GRAS family in *Phyllostachys edulis*. Sci. Rep. 6:27640. 10.1038/srep27640 27325361 PMC4914925

