# Supplementary material for: Evolutionary Analyses of GRAS Transcription Factors in Angiosperms
Source: Front Plant Sci. 2017 Mar 2;8:273. doi: 10.3389/fpls.2017.00273 (PMC5332381; doi:10.3389/fpls.2017.00273)
Supplement: Supplementary Figures 1–40 — Individual phylogenies for orthogroups and subfamilies. [file DataSheet4.DOCX]

Supplementary Material

Evolutionary analyses of GRAS transcription factors in Angiosperms

**Cenci A and Rouard M***

*** Correspondence:** Corresponding Author: a.cenci@cgiar.org

## 1. Supplementary Figures


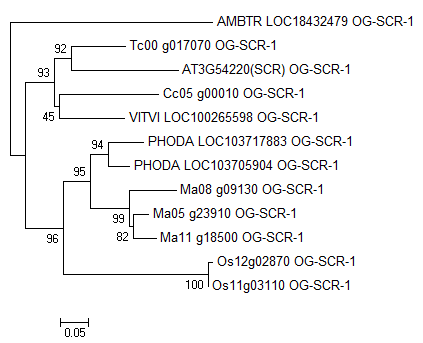


**Supplementary Figure 1.** Phylogenetic tree of OG-SCR-1, based on 490 positions. Amborella sequence was used as outgroup. Branch support is based on % of aLRT.

## 2. Supplementary Figures


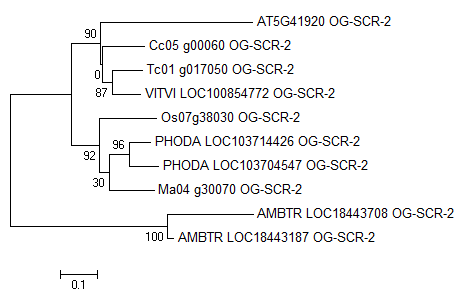


**Supplementary Figure 2.** Phylogenetic tree of OG-SCR-2, based on 356 positions. Amborella sequences was used as outgroup. Branch support is based on % of aLRT.

## 3. Supplementary Figures


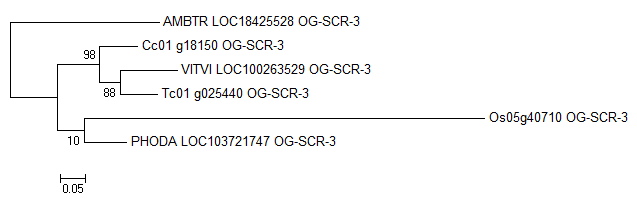


**Supplementary Figure 3.** Phylogenetic tree of OG-SCR-3, based on 345 positions. Amborella sequence was used as outgroup. Branch support is based on % of aLRT.

## 4. Supplementary Figures


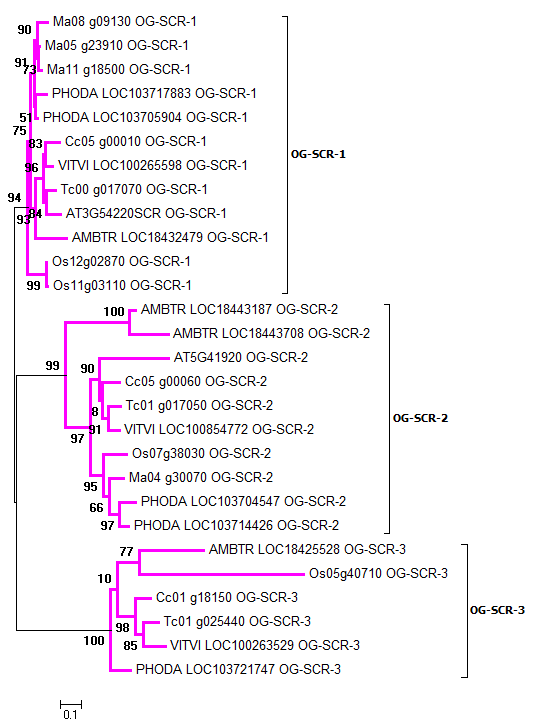


**Supplementary Figure 4.** Unrooted phylogenetic tree of SCR subfamily, based on 329 positions. Branch support is based on % of aLRT.

## 5. Supplementary Figures


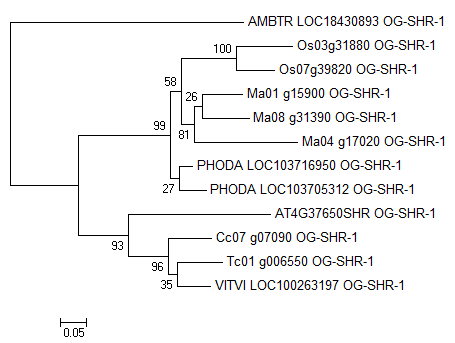


**Supplementary Figure 5.** Phylogenetic tree of OG-SHR-1, based on 398 positions. Amborella sequence was used as outgroup. Branch support is based on % of aLRT.

## 6. Supplementary Figures


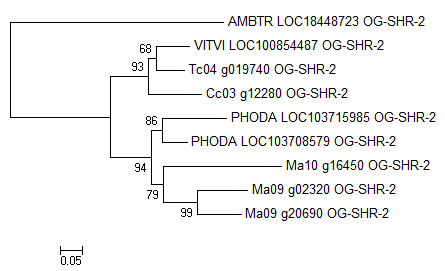


**Supplementary Figure 6.** Phylogenetic tree of OG-SHR-2, based on 380 positions. Amborella sequence was used as outgroup. Branch support is based on % of aLRT.

## 7. Supplementary Figures


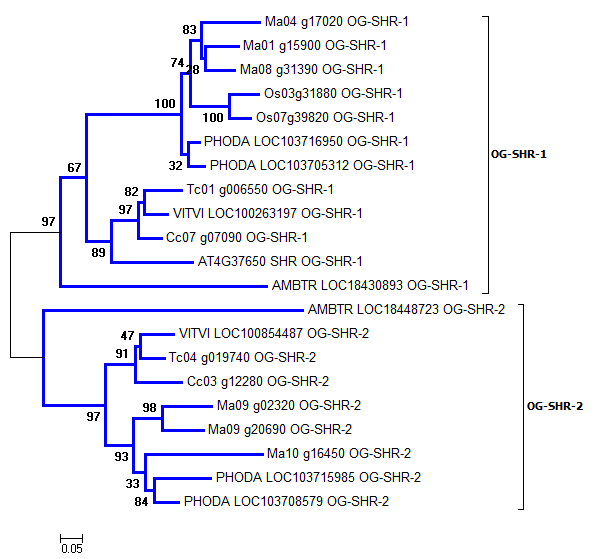


**Supplementary Figure 7.** Unrooted phylogenetic tree of SHR subfamily, based on 334 positions. Branch support is based on % of aLRT.

## 8. Supplementary Figures


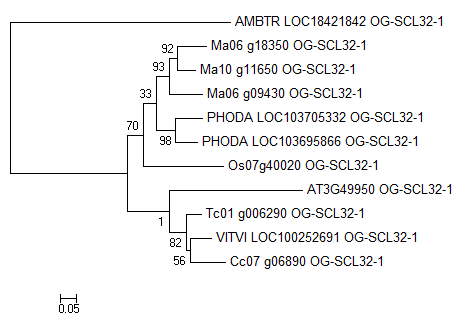


**Supplementary Figure 8.** Phylogenetic tree of OG-SCL32-1, based on 407 positions. Amborella sequence was used as outgroup. Branch support is based on % of aLRT.

## 9. Supplementary Figures


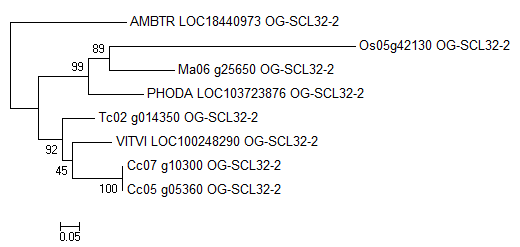


**Supplementary Figure 9.** Phylogenetic tree of OG-SCL32-2, based on 391 positions. Amborella sequence was used as outgroup. Branch support is based on % of aLRT.

## 10. Supplementary Figures


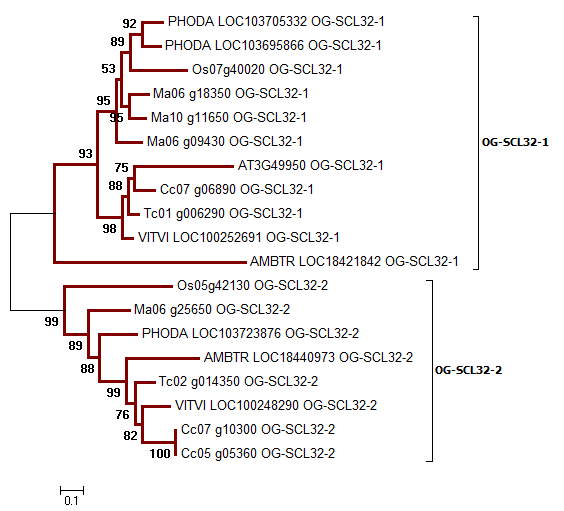


**Supplementary Figure 10.** Unrooted phylogenetic tree of SCL32 subfamily, based on 334 positions. Branch support is based on % of aLRT.

## 11. Supplementary Figures


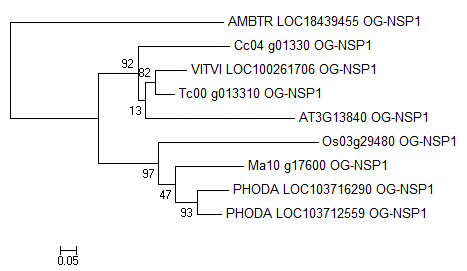


**Supplementary Figure 11** Phylogenetic tree of OG-NSP1, based on 345 positions. Amborella sequence was used as outgroup. Branch support is based on % of aLRT.

## 12. Supplementary Figures


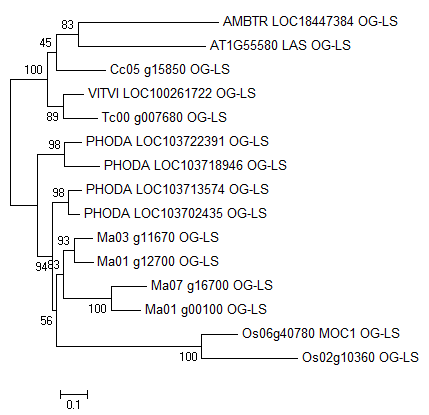


**Supplementary Figure 12.** Phylogenetic tree of OG-LS, based on 307 positions. Amborella sequence was used as outgroup. Branch support is based on % of aLRT.

## 13. Supplementary Figures


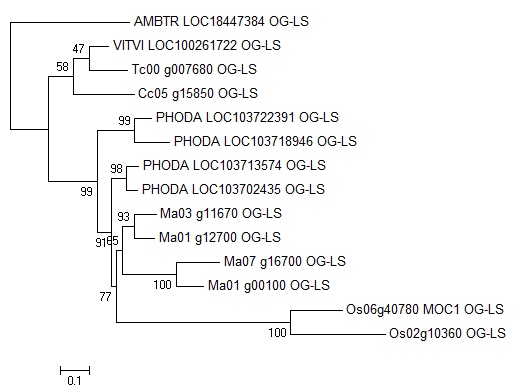


**Supplementary Figure 13.** Phylogenetic tree of OG-LS (without AT1G55580), based on 314 positions. Amborella sequence was used as outgroup. Branch support is based on % of aLRT.

## 14. Supplementary Figures


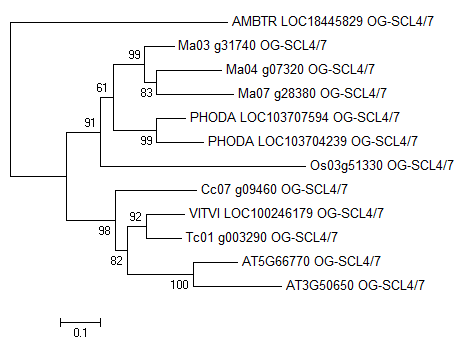


**Supplementary Figure 14.** Phylogenetic tree of OG-SCL4/7, based on 423 positions. Amborella sequence was used as outgroup. Branch support is based on % of aLRT.

## 15. Supplementary Figures


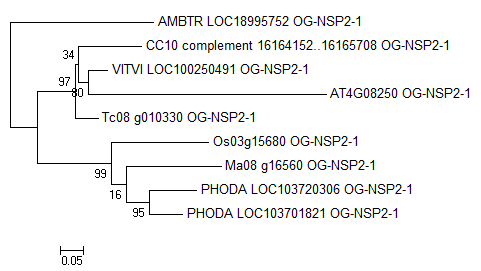


**Supplementary Figure 15.** Phylogenetic tree of OG-NSP2-1, based on 372 positions. Amborella sequence was used as outgroup. Branch support is based on % of aLRT.

## 16. Supplementary Figures


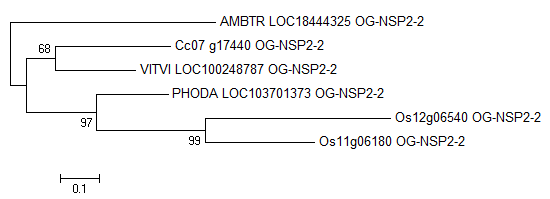


**Supplementary Figure 16.** Phylogenetic tree of OG-NSP2-2, based on 255 positions. Amborella sequence was used as outgroup. Branch support is based on % of aLRT.

## 17. Supplementary Figures


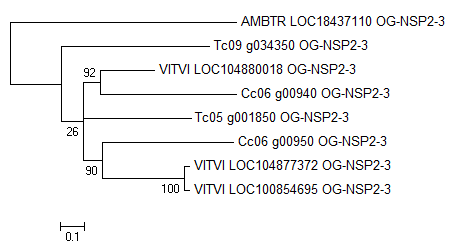


**Supplementary Figure 17.** Phylogenetic tree of OG-NSP2-3, based on 419 positions. Amborella sequence was used as outgroup. Branch support is based on % of aLRT.

## 18. Supplementary Figures


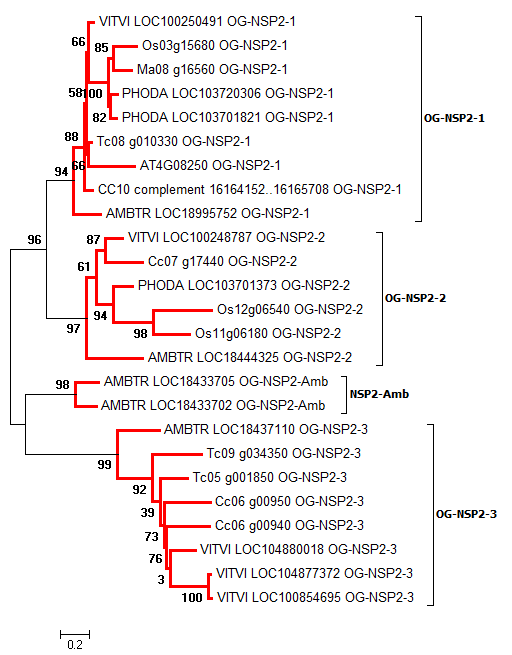


**Supplementary Figure 18.** Unrooted phylogenetic tree of NSP2 subfamily, based on 170 positions. Branch support is based on % of aLRT.

## 19. Supplementary Figures


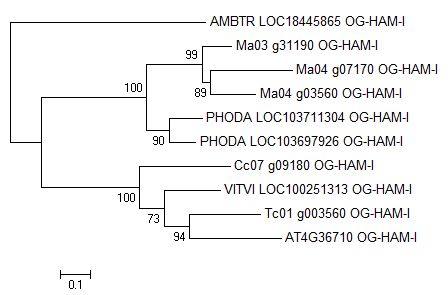


**Supplementary Figure 19.** Phylogenetic tree of OG-HAM-I, based on 343 positions. Amborella sequence was used as outgroup. Branch support is based on % of aLRT.

## 20. Supplementary Figures


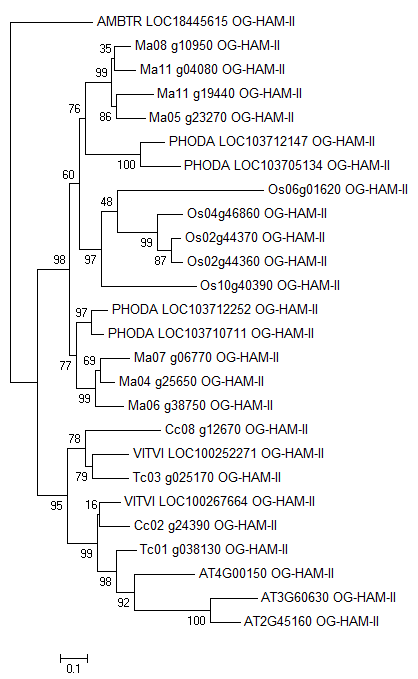


**Supplementary Figure 20.** Phylogenetic tree of OG-HAM-II, based on 271 positions. Amborella sequence was used as outgroup. Branch support is based on % of aLRT.

## 21. Supplementary Figures


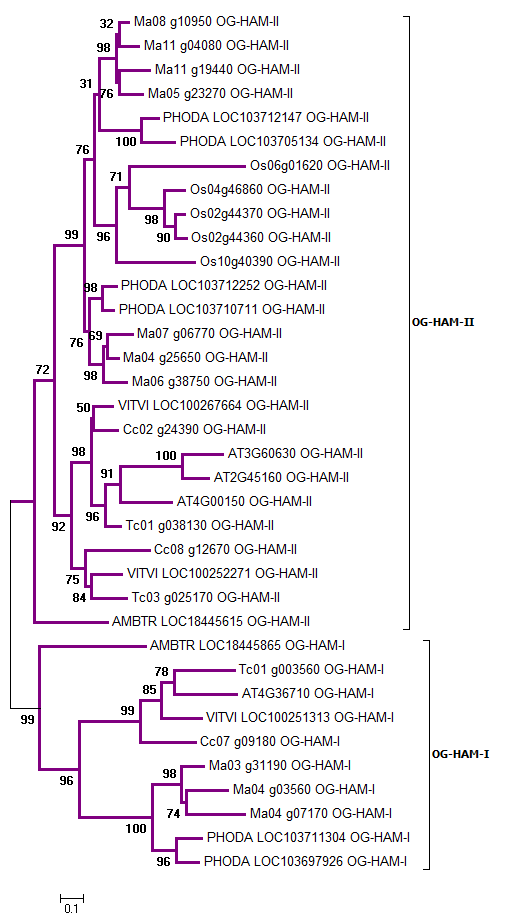


**Supplementary Figure 21.** Unrooted phylogenetic tree of HAM subfamily, based on 251 positions. Branch support is based on % of aLRT.

## 22. Supplementary Figures


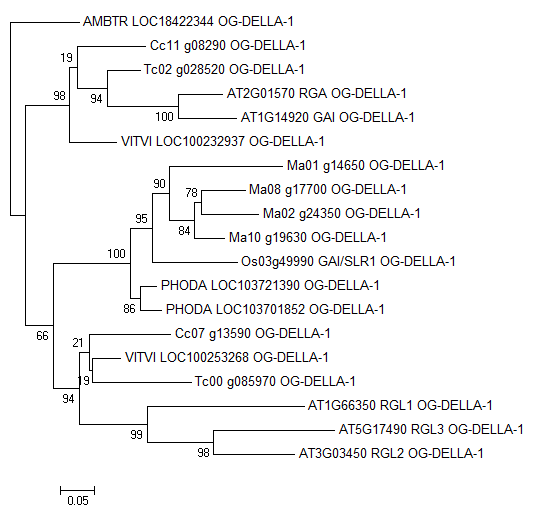


**Supplementary Figure 22.** Phylogenetic tree of OG-DELLA-1, based on 409 positions. Amborella sequence was used as outgroup. Branch support is based on % of aLRT.

## 23. Supplementary Figures


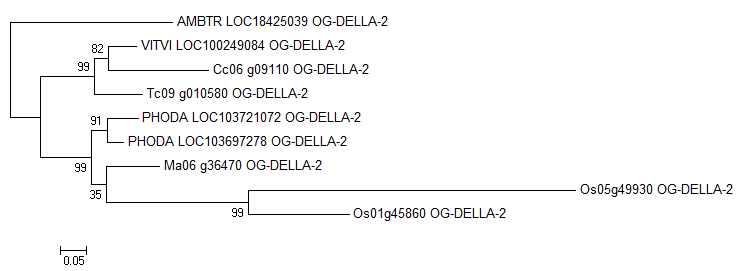


**Supplementary Figure 23.** Phylogenetic tree of OG-DELLA-2, based on 405 positions. Amborella sequence was used as outgroup. Branch support is based on % of aLRT.

## 24. Supplementary Figures


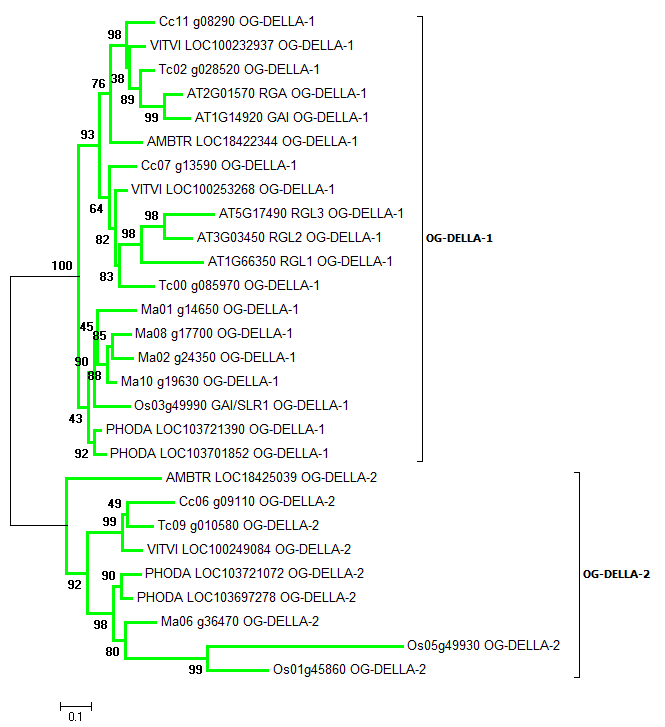


**Supplementary Figure 24.** Unrooted phylogenetic tree of DELLA subfamily, based on 364 positions. Branch support is based on % of aLRT.

## 25. Supplementary Figures


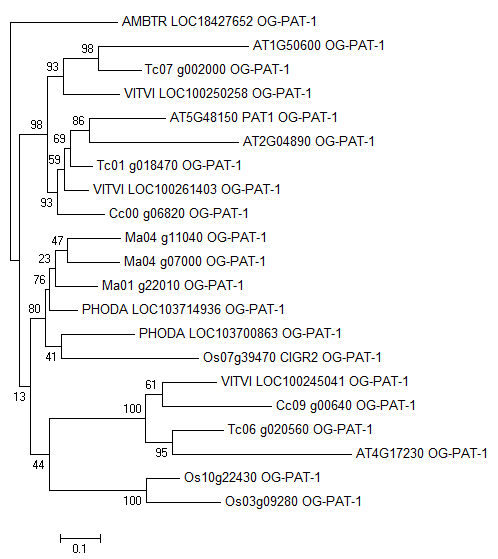


**Supplementary Figure 25.** Phylogenetic tree of OG-PAT-1, based on 434 positions. Amborella sequence was used as outgroup. Branch support is based on % of aLRT.

## 26. Supplementary Figures


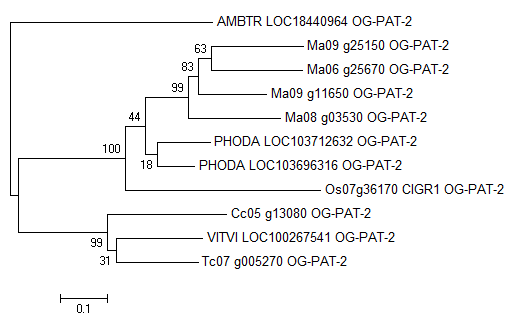


**Supplementary Figure 26.** Phylogenetic tree of OG-PAT-2, based on 519 positions. Amborella sequence was used as outgroup. Branch support is based on % of aLRT.

## 27. Supplementary Figures


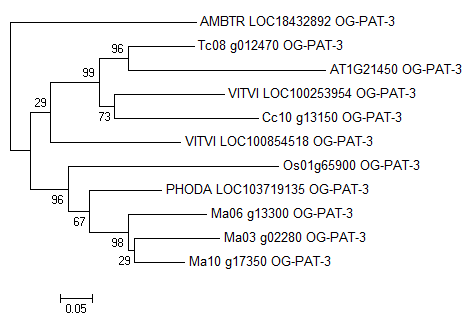


**Supplementary Figure 27.** Phylogenetic tree of OG-PAT-3, based on 479 positions. Amborella sequence was used as outgroup. Branch support is based on % of aLRT.

## 28. Supplementary Figures


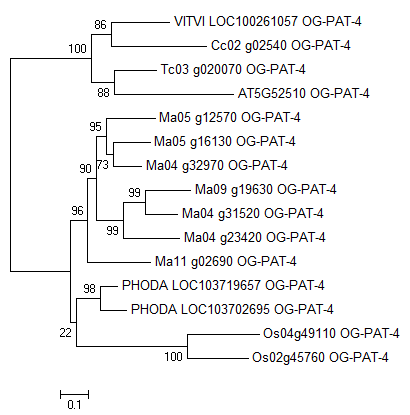


**Supplementary Figure 28.** Phylogenetic tree of OG-PAT-4, based on 427 positions. Amborella sequence was used as outgroup. Branch support is based on % of aLRT.

## 29. Supplementary Figures


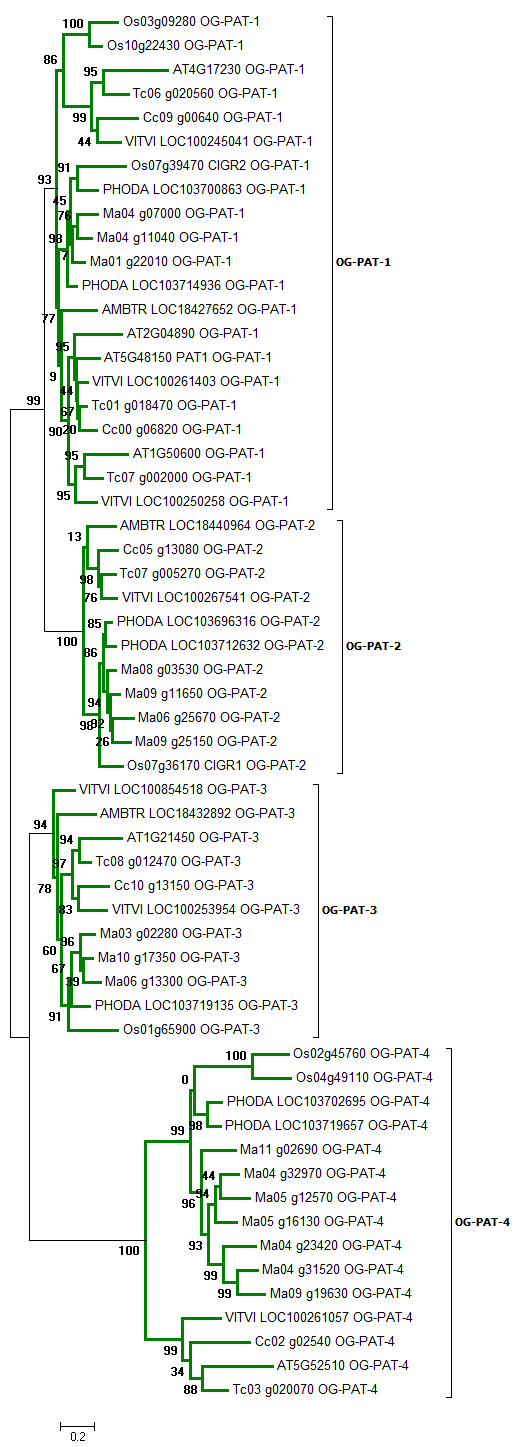


**Supplementary Figure 29.** Unrooted phylogenetic tree of PAT subfamily, based on 351 positions. Branch support is based on % of aLRT.

## 30. Supplementary Figures


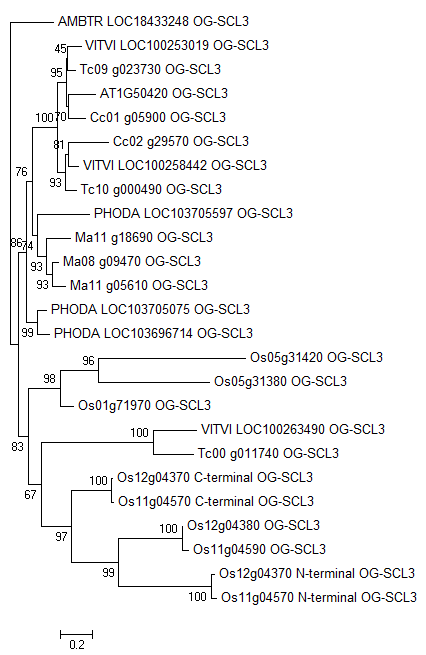


**Supplementary Figure 30.** Phylogenetic tree of OG-SCL3, based on 310 positions. Amborella sequence was used as outgroup. Branch support is based on % of aLRT.

## 31. Supplementary Figures


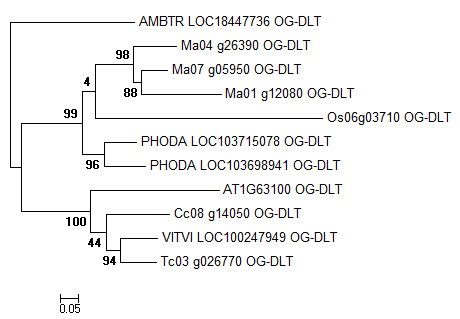


**Supplementary Figure 31.** Phylogenetic tree of OG-DLT, based on 496 positions. Amborella sequence was used as outgroup. Branch support is based on % of aLRT.

## 32. Supplementary Figures

**
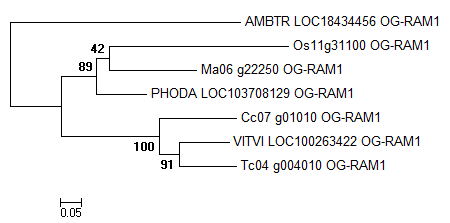
**

**Supplementary Figure 32.** Phylogenetic tree of OG-RAM1, based on 520 positions. Amborella sequence was used as outgroup. Branch support is based on % of aLRT.

## 33. Supplementary Figures


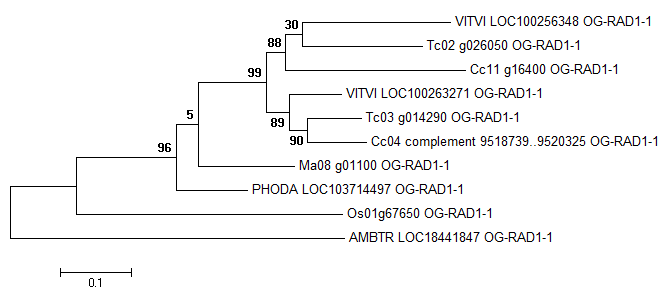


**Supplementary Figure 33.** Phylogenetic tree of OG-RAD1-1, based on 377 positions. Amborella sequence was used as outgroup. Branch support is based on % of aLRT.

## 34. Supplementary Figures


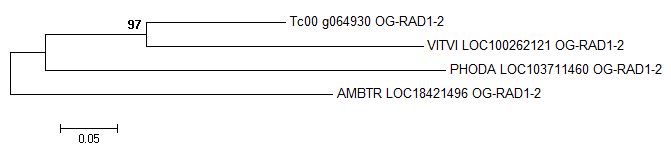


**Supplementary Figure 34.** Phylogenetic tree of OG-RAD1-2, based on 466 positions. Amborella sequence was used as outgroup. Branch support is based on % of aLRT.

## 35. Supplementary Figures


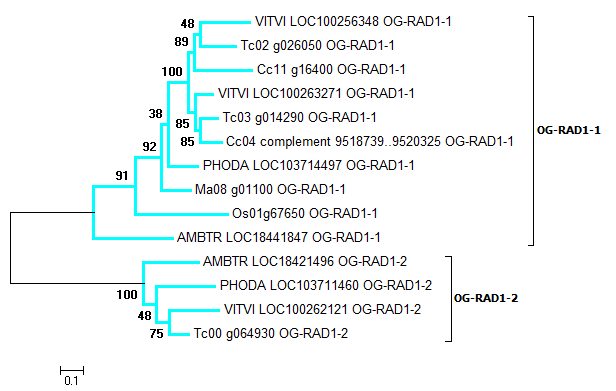


**Supplementary Figure 35.** Unrooted phylogenetic tree of RAD1 subfamily, based on 368 positions. Branch support is based on % of aLRT.

## 36. Supplementary Figures


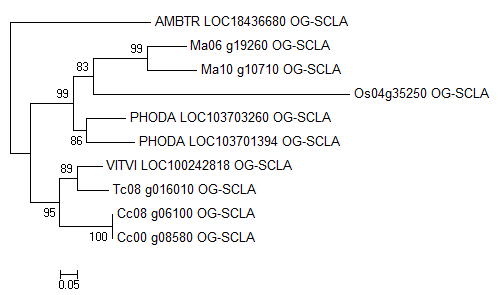


**Supplementary Figure 36.** Phylogenetic tree of OG-SCLA, based on 347 positions. Amborella sequence was used as outgroup. Branch support is based on % of aLRT.

## 37. Supplementary Figures


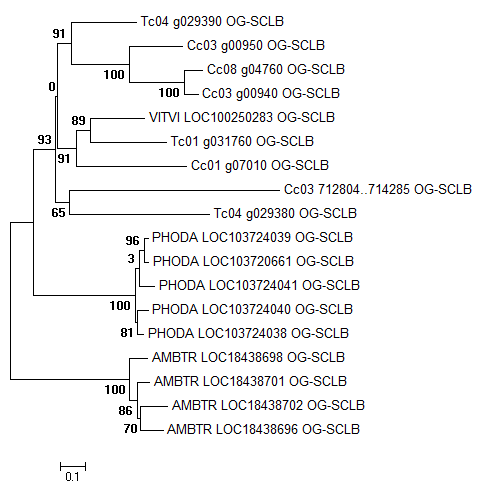


**Supplementary Figure 37.** Phylogenetic tree of OG-SCLB, based on 400 positions. Amborella sequence was used as outgroup. Branch support is based on % of aLRT.

## 38. Supplementary Figures


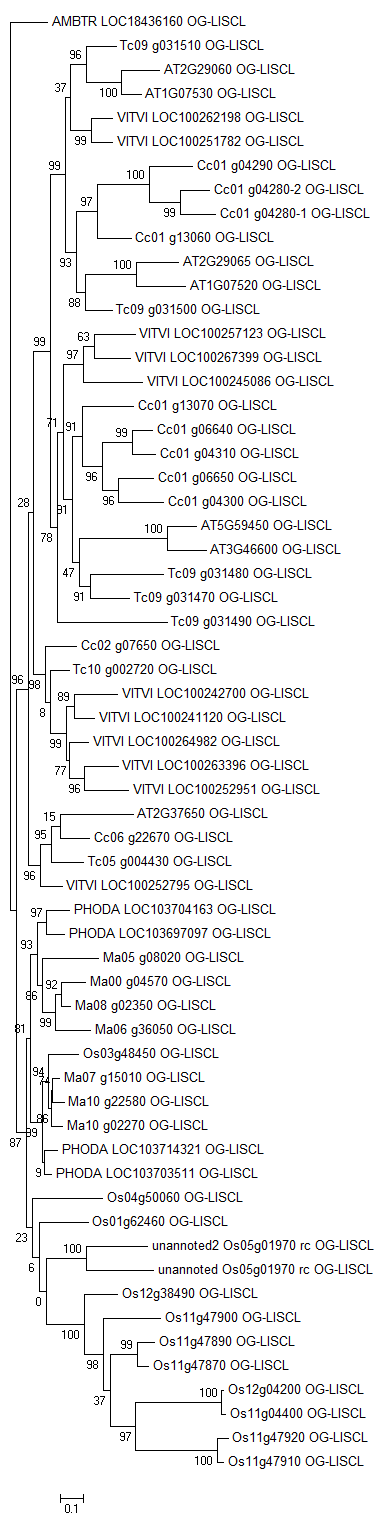


**Supplementary Figure 38.** Phylogenetic tree of OG-LISCL, based on 361 positions. Amborella sequence was used as outgroup. Branch support is based on % of aLRT.

## 39. Supplementary Figures


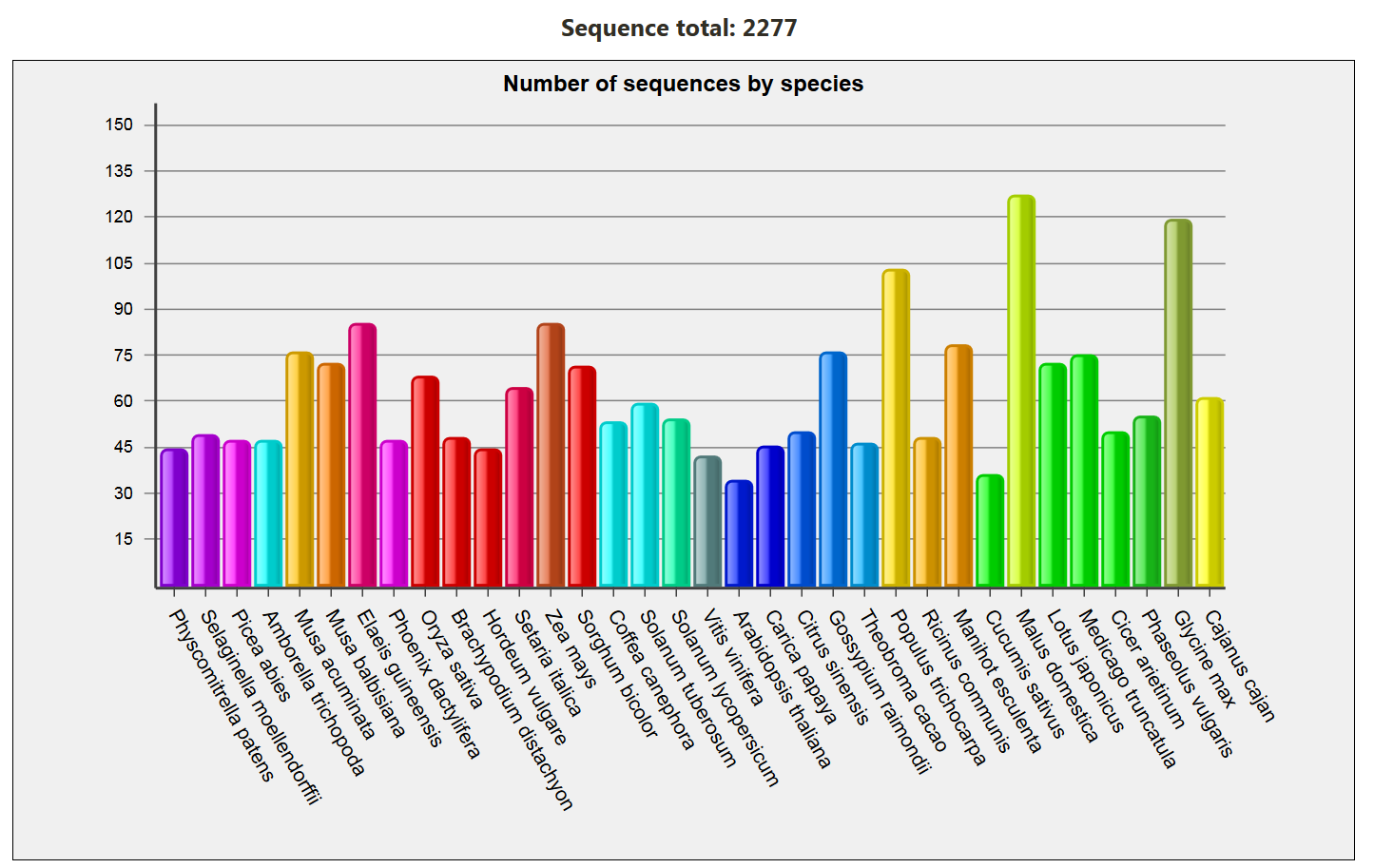


**Supplementary Figure 39.** Number of GRAS genes annotated in plant species (GreenPhyl database).( <http://www.greenphyl.org/cgi-bin/family.cgi?p=id&family_id=62#tab-famcomp>)

## 40. Supplementary Figures


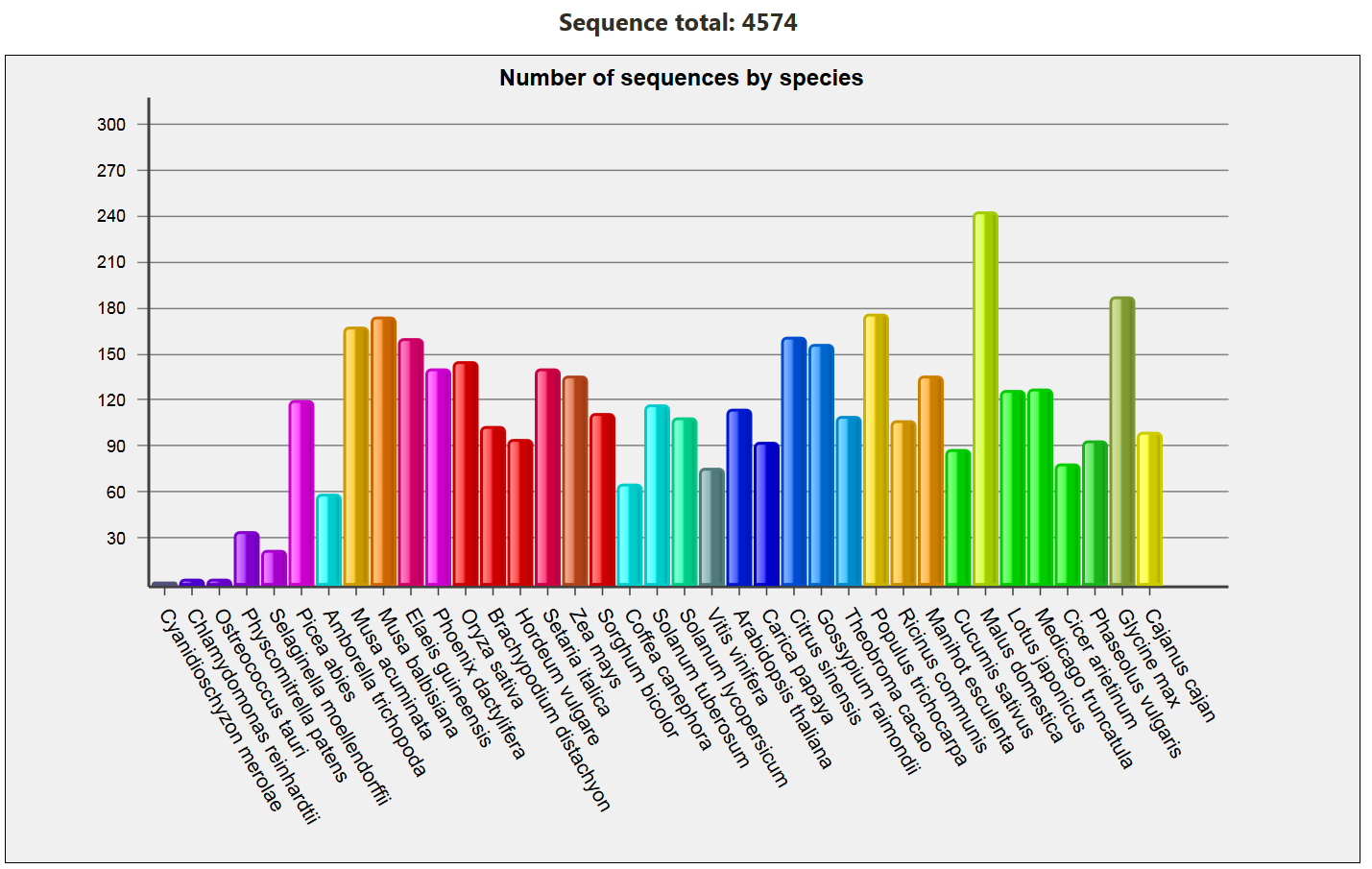


**Supplementary Figure 40.** Number of NAC genes annotated in plant species (GreenPhyl database).(<http://www.greenphyl.org/cgi-bin/family.cgi?p=id&family_id=20#tab-famcomp>)
